# A novel serine protease inhibitor as potential treatment for dry eye syndrome and ocular inflammation

**DOI:** 10.1038/s41598-020-74159-w

**Published:** 2020-10-14

**Authors:** Cedric Joossen, Adrienn Baán, Carlos Moreno-Cinos, Jurgen Joossens, Nathalie Cools, Ellen Lanckacker, Lieve Moons, Kim Lemmens, Anne-Marie Lambeir, Erik Fransen, Peter Delputte, Guy Caljon, Pieter Van Der Veken, Louis Maes, Ingrid De Meester, Filip Kiekens, Koen Augustyns, Paul Cos

**Affiliations:** 1 Laboratory of Microbiology, Parasitology and Hygiene (LMPH), Faculty of Pharmaceutical, Biomedical and Veterinary Sciences, University of Antwerp, Universiteitsplein 1, 2610 Wilrijk, Belgium; 2 Laboratory of Pharmaceutical Technology and Biopharmacy, Faculty of Pharmaceutical, Biomedical and Veterinary Sciences, University of Antwerp, Universiteitsplein 1, 2610 Wilrijk, Belgium; 3 Laboratory of Medicinal Chemistry, Faculty of Pharmaceutical, Biomedical and Veterinary Sciences, University of Antwerp, Universiteitsplein 1, 2610 Wilrijk, Belgium; 4 Laboratory of Experimental Hematology, Vaccine & Infectious Disease Institute (VAXINFECTIO), Faculty of Medicine and Health Sciences, University of Antwerp, Universiteitsplein 1, 2610 Wilrijk, Belgium; 5 Neural Circuit Development and Regeneration Research Group, Faculty of Biology, University of Leuven, Naamsestraat 61, Leuven, Belgium; 6 Laboratory of Medical Biochemistry, Faculty of Pharmaceutical, Biomedical and Veterinary Sciences, University of Antwerp, Universiteitsplein 1, 2610 Wilrijk, Belgium; 7 Laboratory of Medical Genetics, Faculty of Pharmaceutical, Biomedical and Veterinary Sciences, University of Antwerp, Universiteitsplein 1, 2610 Wilrijk, Belgium

**Keywords:** Proteases, Drug development

## Abstract

Dry eye syndrome (DES), a multifactorial disorder which leads to ocular discomfort, visual disturbance and tear film instability, has a rising prevalence and limited treatment options. In this study, a newly developed trypsin-like serine protease inhibitor (UAMC-00050) in a tear drop formulation was evaluated to treat ocular inflammation. A surgical animal model of dry eye was employed to investigate the potential of UAMC-00050 on dry eye pathology. Animals treated with UAMC-00050 displayed a significant reduction in ocular surface damage after evaluation with sodium fluorescein, compared to untreated, vehicle treated and cyclosporine-treated animals. The concentrations of IL-1α and TNF-α were also significantly reduced in tear fluid from UAMC-00050-treated rats. Additionally, inflammatory cell infiltration in the palpebral conjunctiva (CD3 and CD45), was substantially reduced. An accumulation of pro-MMP-9 and a decrease in active MMP-9 were found in tear fluid from animals treated with UAMC-00050, suggesting that trypsin-like serine proteases play a role in activating MMP-9 in ocular inflammation in this animal model. Comparative qRT-PCR analyses on ocular tissue indicated the upregulation of tryptase, urokinase plasminogen activator receptor (uPAR) and protease-activated receptor 2 (PAR2). The developed UAMC-00050 formulation was stable up to 6 months at room temperature in the absence of light, non-irritating and sterile with compatible pH and osmolarity. These results provide a proof-of-concept for the in vivo modifying potential of UAMC-00050 on dry eye pathology and suggest a central role of trypsin-like serine proteases and PAR2 in dry eye derived ocular inflammation.

## Introduction

Dry eye syndrome (DES) or keratoconjunctivitis sicca (KCS) is a multifactorial disease of the ocular surface characterized by a loss of homeostasis of the tear film, and accompanied by ocular symptoms in which tear film instability and hyperosmolarity ocular surface inflammation and damage, and neurosensory abnormalities play etiological roles (TFOS DEWS II)^1^. DES is strongly related to age but its prevalence in the younger population is rising due to increased contact lens wear, prolonged time spent using computers and smartphones, low air quality and low humidity^2^. DES-derived eye irritation, photophobia, blurred and fluctuating vision can have a severe negative impact on patients’ quality of life. Various components of the lacrimal functional unit can be affected, resulting in dysfunction of the ocular tear film. DES can be classified into three types: (a) tear-deficient dry eye (b) evaporative dry eye^2^ and (c) short tear film breakup time (TFBUT)-type dry eye (unstable tear film)^3^. All forms of DES lead to osmotic/cellular stress at the ocular surface. This initiates deregulation of ocular surface homeostasis and the establishment of a pro-inflammatory environment. Exposure of ocular epithelial cells to elevated tear osmolarity and mechanical stress activates stress-associated inflammatory pathways, such as mitogen-activated protein kinase (MAPK) and particularly p38 and c-Jun N-terminal kinases (JNK), together with the activation of Nuclear factor Kappa Beta (NF-κβ)^2,4–8^. Activation of these pathways results in the transcription of stress-related genes, including inflammatory cytokines such as tumor necrosis factor (TNF)-α, Interleukin (IL)-1 and IL-6, as well as matrix metalloproteinases (MMPs), chemokines and intracellular adhesion molecules (ICAMs)^2,7,9,10^. All these inflammatory mediators promote the activation of immature antigen presenting cells (APCs) and induce their migration to draining lymph nodes. APCs can be responsible for priming naïve T cells in lymphoid tissue and lead to the expansion of autoreactive CD4^+^ helper cells (Th) Th1 and Th17 subsets. These primed T cells subsequently infiltrate the ocular surface, where they secrete additional pro-inflammatory cytokines^2,4,11,12^. Some of these cytokines, like IFN-γ, promote the production of chemokines, chemokine receptors and cell adhesion molecules (CAMs) that facilitate the ingress of pathogenic immune cells^13^. MMPs, such as MMP-9, degrade the extracellular matrix and destroy tight junctions between corneal epithelial cells, impairing ocular surface integrity and facilitating inflammatory cell infiltration^2,14,15^. Thus, chronic tear film dysfunction leads to a self-perpetuating inflammatory cycle which affects the integrity of the ocular surface and ocular function.

Artificial tear substituents are the most commonly applied therapy for dry eye and mild forms of ocular inflammation. Despite bringing momentary relief by dilution of the inflammatory markers present in the tear fluid and briefly lowering tear osmolarity, artificial tears have no anti-inflammatory properties and do not address the underlying pathogenesis of DES. Until recently, Cyclosporin A (CyA), a fungal derived peptide with immunosuppressive properties (Restasis), was the only long-term FDA-approved local anti-inflammatory treatment for DES^16^. Although CyA has been shown to decrease DES-symptoms in patients, it has also been reported that a relatively large percentage of patients have an incomplete response to CyA or have significant side effects^16^. In July 2016, Lifitegrast (Xiidra), a small molecule competitive lymphocyte function-associated antigen-1 (LFA-1) antagonist was approved by the FDA for the treatment of DES. This new compound showed promising results in multiple randomized, prospective clinical trials^17,18^. Finally, topical corticosteroids are only suitable for short periods of time during exacerbations of inflammation, but they are not suitable for long-term treatment of DES due to many adverse effects^19^.

Serine proteases play a pivotal role in several inflammatory conditions and have come in the spotlight as therapeutic targets for many diseases with an inflammatory component like keratitis, arthritis, vasculitis, asthma, cancer, multiple sclerosis and fibrosis^20–29^. They have the ability to promote inflammatory protein expression and have a direct influence on the degradation of extracellular matrix components, loss of epithelial barrier function and MMP-9 activation^20–24^. Because these pathological processes also occur in DES-associated inflammation, we hypothesize the involvement of certain serine proteases in this disorder.

**Figure 1:**
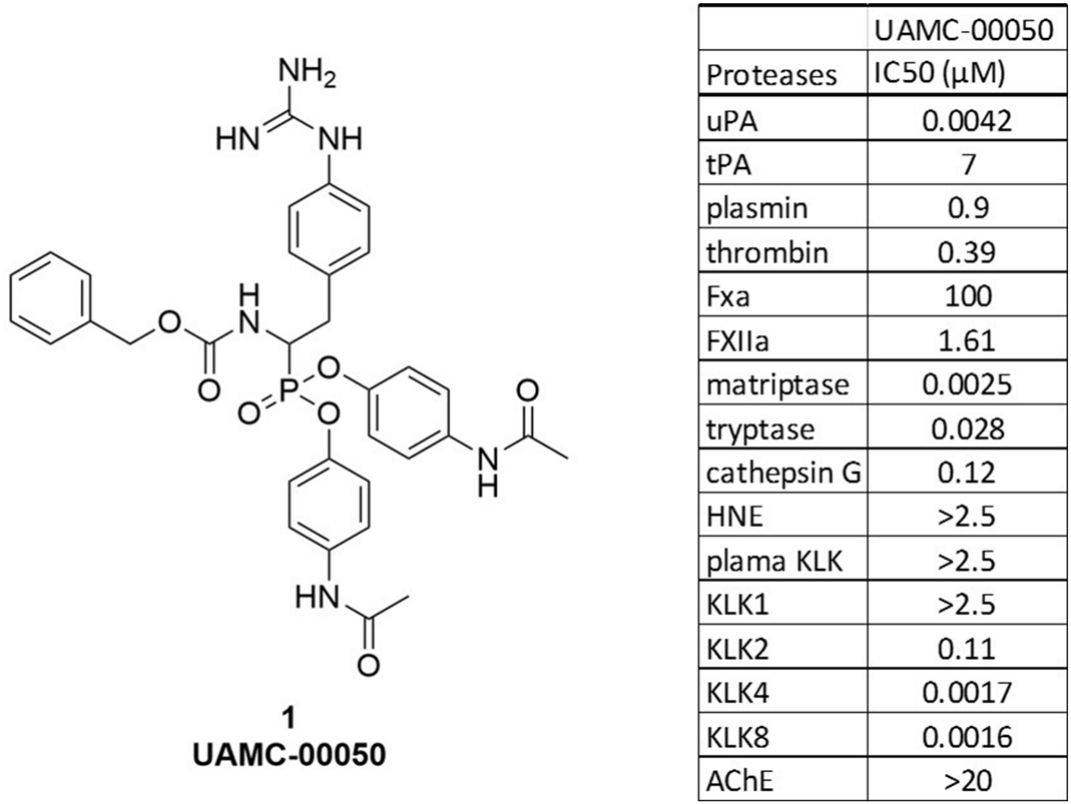
Chemical structure and inhibitory profile of UAMC-00050–Laboratory of Medicinal Chemistry, University of Antwerp, 2016. uPA: urokinase plasminogen activator, tPA: tissue plasminogen activator, Fxa: Factor Xa, FXIIa: factor XIIa, HNE: Human neutrophil elastase, KLK: kallikrein, AchE: acetylcholinesterase.

Peptide-derived diphenyl phosphonates irreversible serine protease inhibitors form a covalent bond with the active site serine side chain hydroxyl group. The selectivity profile of this type of compounds is usually enforced by incorporating peptidic tails. In contrast, Joossens et al*.* demonstrated that potent and selective inhibition can also be achieved with small, non-peptidic diphenyl phosphonates^30–32^. This approach was confirmed by Mucha et al.^33^. In this study UAMC-00050 (Fig. 1), a small diphenyl phosphonate developed at the laboratory of Medicinal Chemistry at the University of Antwerp^30–32^ was evaluated. This compound was characterized as a serine protease inhibitor with a well-defined multi-target inhibition profile described by Joossens et al.^31^ and Ceuleers et al.^34^ (Fig. 1). Several of the inhibited trypsin-like serine proteases such as kallikreins, tryptase, thrombin, cathepsin G, matriptase and urokinase plasminogen activator (uPA) are known to activate protease-activated receptors (PARs), and are directly or indirectly involved in many inflammatory and extracellular matrix degrading processes^20,35–42^. As these processes feature prominently in the disease progression of DES, we hypothesized that this multi-target inhibitor could become a potential treatment. As the compound did not show any signs of cytotoxicity, it was evaluated after local application in a tear drop formulation in a DES animal model^43^. The tear drop formulation was specifically developed to preserve the chemical and physical stability of the serine protease inhibitor to safeguard its longer shelf-life.

## Results

### Tear volume

A significant tear volume reduction of approximately 50% was observed in dry eyes compared to control eyes (p < 0.001). Although a positive trend was observed for CyA and UAMC-00050, these results were not statistically significant.

### Fluorescein score

**Figure 2:**
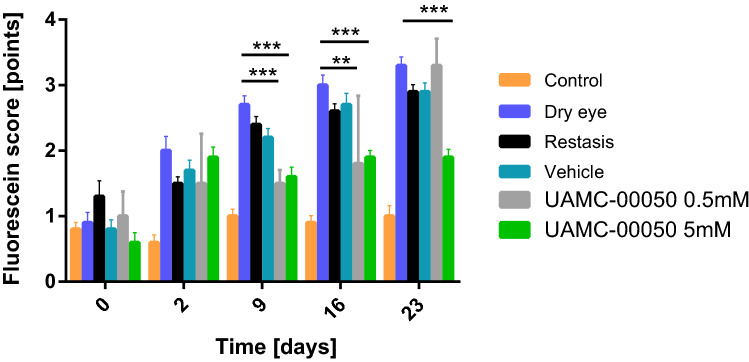
Fluorescein staining of control eyes, untreated dry eyes, CyA, vehicle and UAMC-00050 5 mM and 0.5 mM treated eyes (23 days). Data represent mean values ± SD of 4 experiments (N = 18 over 4 experiments), except for UAMC-00050 0.5 mM, where N = 5 (1 experiment).

A significant effect on tissue damage was registered as from 9 days of treatment onwards. UAMC-00050 5 mM treated animals had the lowest overall fluorescein scores of all treatments and it was the only treatment with a statistically significant effect (p < 0.001) for every time point ≥ 9 days (Fig. 2). Initially, UAMC-00050 at a lower concentration of 0.5 mM had a statistically significant effect on ocular surface damage. This effect declined after 23 days of treatment, indicating a strong dose-dependent effect. CyA and vehicle treated animals displayed no significant effect on ocular surface damage (Fig. 2). Additionally, a statistically significant difference in ocular surface damage between UAMC-00050 and vehicle treated animals was registered for every time point after 2 days (at day 9 p = 0.024, day 16 p = 0.002 and at day 23 p < 0.001).

### Levels of pro-inflammatory cytokines in tear fluid

#### IL-1α

**Figure 3:**
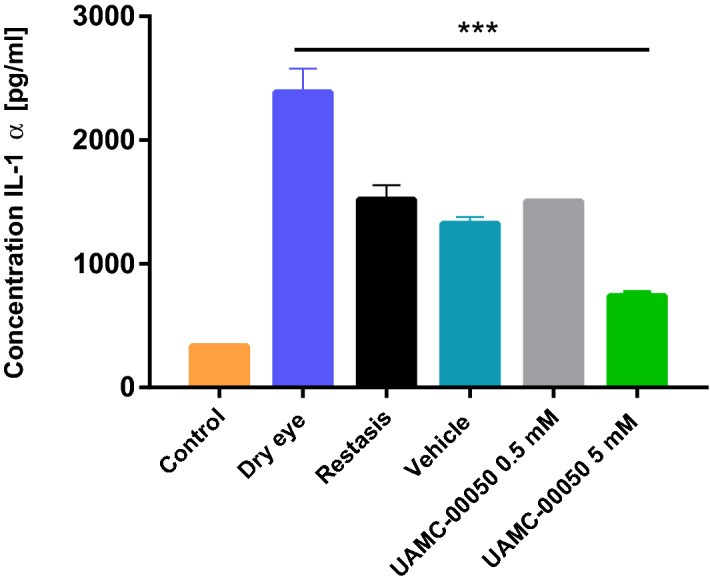
Mean IL-1α concentration [pg ml^−1^] of control eyes, untreated dry eyes, CyA, vehicle and UAMC-00050 0.5 and 5 mM treated eyes. Data represent mean values ± SD of 4 experiments, where N in every experiment was 1 pool from 4 to 5 animals except for UAMC 0.5 mM, where N = 1 pool from 1 experiment, using 5 animals (N = 18 for every experimental group except UAMC-00050 0.5 mM where N = 5) .

From day 7 onwards, 5 mM UAMC-00050-treated animals clearly displayed lowered IL-1α concentrations compared to vehicle and CyA treated animals, highlighting the potential of UAMC-00050. UAMC-00050-treated animals had the lowest overall IL-1α concentrations at every time point. A vehicle-effect was also observed, indicating that ocular wetting does lower IL-1α levels compared to untreated dry eye in this animal model (Fig. 3). This effect was not statistically relevant however.

Mean overall IL-1α concentrations indicated a statistically significant effect for UAMC-00050 5 mM treated animals (p < 0.001). UAMC-00050 0.5 mM and CyA had no statistically relevant effect (Fig. 3).

#### TNF-α

**Figure 4:**
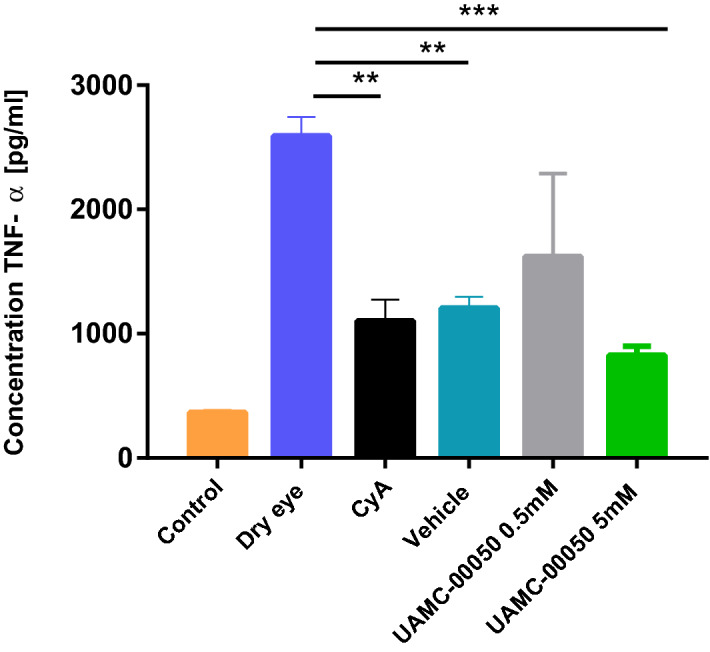
Mean TNF-α concentration [pg ml^−1^] of control eyes, untreated dry eyes, CyA, vehicle and UAMC-00050 0.5 mM and 5 mM treated eyes. Data represent mean values ± SD of 4 experiments (N = 18 for every experimental group except UAMC-00050 0.5 mM where N = 5) .

5 mM UAMC-00050-treated animals had the lowest overall TNF-α concentration (Fig. 4). CyA (p = 0.003), vehicle (p = 0.002) and UAMC-00050 (p < 0.001) all had a statistically significant effect on overall tear TNF-α concentrations. Remarkably, although treatment with UAMC-00050 showed the lowest overall TNF-α levels, vehicle treated animals also displayed significantly reduced TNF-α concentrations.

Interestingly, tear substituents (vehicle) succeeded in lowering cytokine concentrations, but they were not effective at lowering ocular surface damage.

### Immunohistochemistry

#### CD45

**Figure 5:**
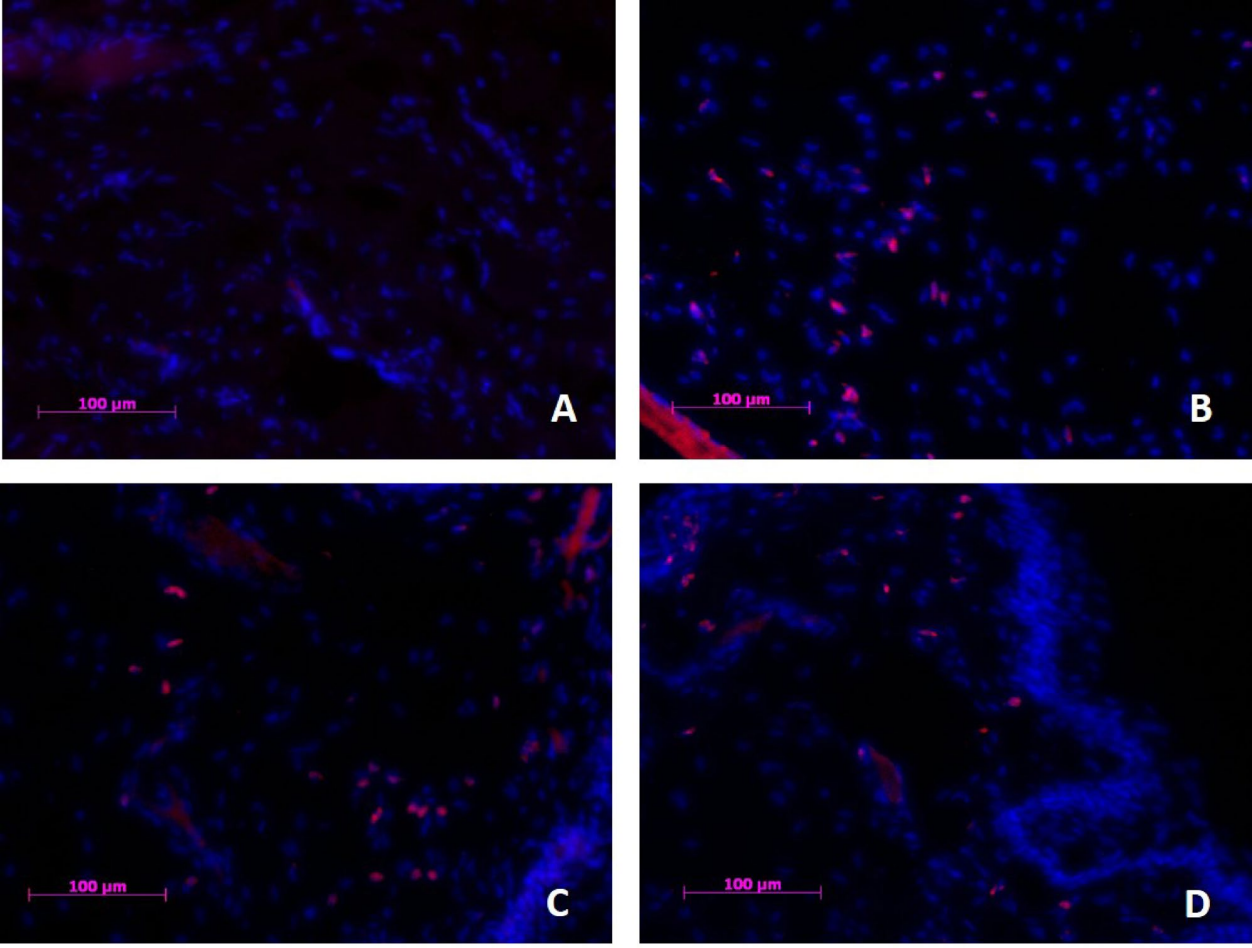
CD45^+^ cell infiltration in palpebral conjunctival tissue of (**A**) healthy control, (**B**) Untreated dry eye, (**C**) Vehicle treated and (**D**) UAMC-00050 treated animals. Eight animals per group were immunohistochemically evaluated and representative images of the respective groups were selected. Blue: Nuclear counterstain (DAPI), Red: CD45^+^ cells. Magnification ×200.

CD45^+^ cells were present, but were scarce in control tissue [2.4 ± 3.3] (Fig. 5A). A lot of CD45^+^ cells were observed in untreated dry eyes [48 ± 9.9] (Fig. 5B) (p < 0.001) and vehicle treated eyes (p = 0.001) (Fig. 5C). UAMC-00050 treated eyes (Fig. 5D) still exhibited an intermediate number [22 ± 10] of CD45^+^ cells (p = 0.003) compared to control. However, a statistically significant difference in CD45^+^ cell count between UAMC-00050 [22 ± 10] and vehicle treated eyes [45 ± 2.5] was observed (p = 0.017).

#### CD3

**Figure 6:**
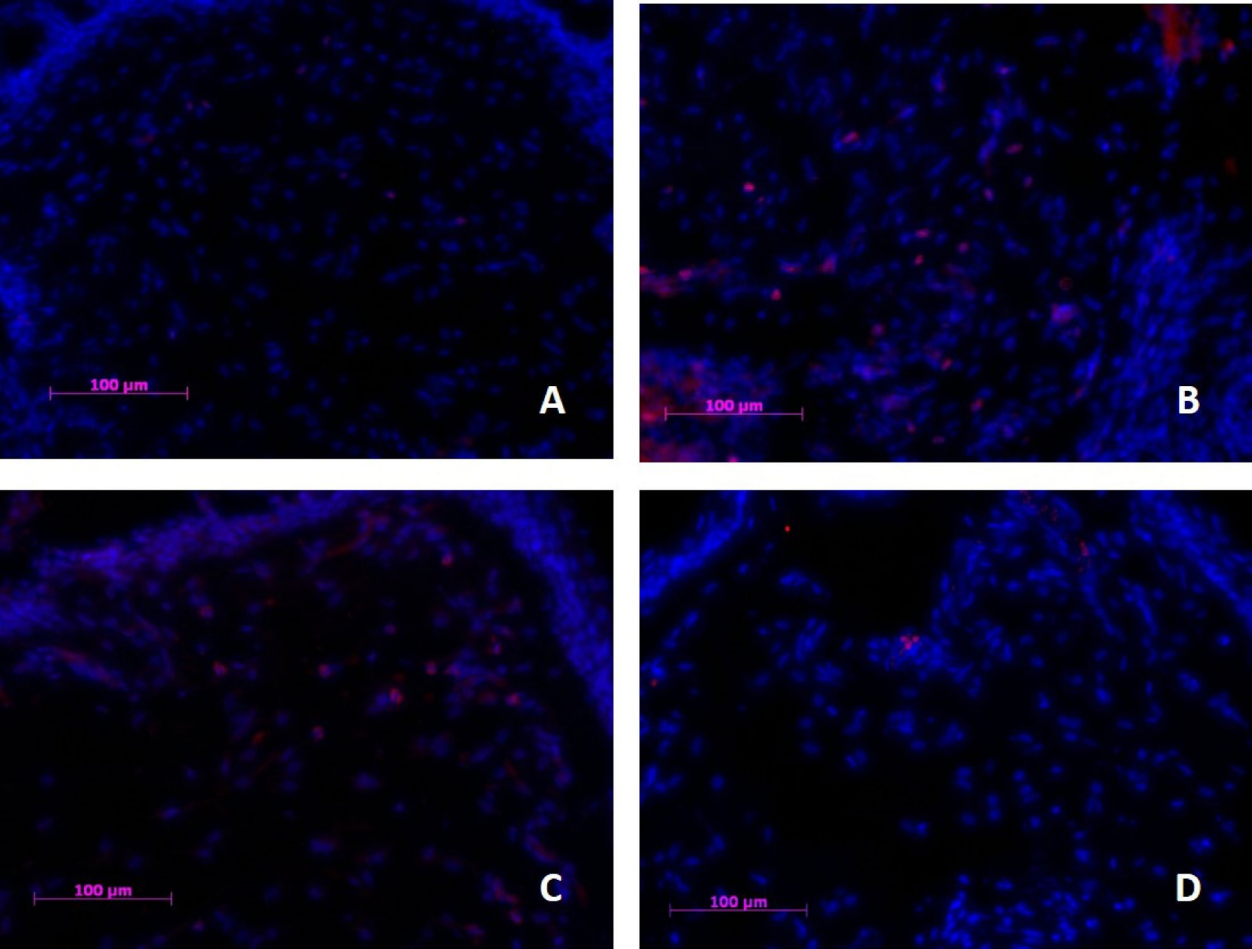
CD3 cell infiltration in palpebral conjunctival tissue of (**A**) healthy control, (**B**) Untreated dry eye, (**C**) Vehicle treated and (**D**) UAMC-00050 treated animals. Eight animals per group were immunohistochemically evaluated and representative images of the respective groups were selected. Blue: Nuclear counterstain (DAPI), Pink: CD3^+^ cells. Magnification  ×200.

Very few CD3^+^ cells were observed in conjunctival tissue of control eyes [1.8 ± 2.7] (Fig. 6A). Untreated [30 ± 3.0] (Fig. 6B) (p = 0.008) and vehicle treated [28 ± 3.3] (Fig. 6C) (p = 0.004) animals with DES displayed distinctively more cells of this type, whereas UAMC-00050 treated animals (Fig. 6D) had substantially less CD3^+^ cells present [7.4 ± 4.1] (no statistical difference from control tissue).

A statistically significant difference in CD3^+^ cell count between UAMC-00050 treated animals and vehicle treated animals (p = 0.035) was found.

### qRT-PCR

#### Palpebral + bulbar conjunctiva

The expression of a number of relevant serine proteases and inflammatory cytokines was investigated in samples of palpebral and conjunctival tissue (Table 1). Both inflammatory expression products (IL-1α, and IL-17A with p = 0.030 and p = 0.004) were upregulated in untreated dry eye, compared to control. Interestingly, control, UAMC-00050 and vehicle treated animals expressed similar levels of IL-1α, despite the observation during tear fluid analysis (Fig. 4) of substantially less IL-1α protein in tear fluid from UAMC-00050 treated animals, compared to vehicle treated animals. The expression of IL-17A was also significantly increased in untreated dry eye. Treatment with UAMC-00050 lowered expression of IL-17A. However, this effect did not statistically differ from the vehicle. Expression of uPAR (p = 0.032), and tryptase (p = 0.026) was significantly increased in conjunctival tissue of animals with DES. Treatment with UAMC-00050 or vehicle did not have an effect on expression of these genes. PAR2 tended to increase in conjunctival tissue but this was not statistically significant. Matriptase did not seem to have an increased expression during DES in conjunctival tissue.

**Table 1:** Mean expression of target gene to endogenous control (GAPDH and ACTB) ± SD, relative to control.

Target	Control	Dry eye	UAMC-00050	Vehicle
**Palpebral + bulbar conjunctiva**				
IL-1α	1.00 ± 0.29	1.50 ± 0.49*	0.95 ± 0.43	0.96 ± 0.24
IL-17A	1.00 ± 1.38	4.12 ± 2.20**	0.21 ± 1.50	0.50 ± 1.70
Tryptase	1.00 ± 0.55	1.62 ± 0.44*	1.61 ± 0.64	1.66 ± 0.52*
Matriptase	1.00 ± 0.10	0.98 ± 0.15	1.01 ± 0.21	1.11 ± 0.29
PAR2	1.00 ± 0.06	1.18 ± 0.39	1.10 ± 0.20	1.05 ± 0.26
uPAR	1.00 ± 0.35	1.40 ± 0.32*	1.80 ± 0.16**	2.83 ± 0.25**
**Cornea**				
Tryptase	1.00 ± 0.50	1.59 ± 0.52*	1.58 ± 0.75	2.68 ± 1.01**
Matriptase	1.00 ± 0.19	1.15 ± 0.16	0.89 ± 0.25	0.91 ± 0.23
PAR2	1.00 ± 0.27	1.28 ± 0.19*	1.22 ± 0.21	1.23 ± 0.15
uPAR	1.00 ± 0.47	1.22 ± 0.52	1.65 ± 0.40*	1.47 ± 0.45

N = 8, comparative qRT-PCR.

*p < 0.05, **p < 0.01 versus control.

#### Cornea

Only the expression of serine proteases was investigated in corneal tissue, as conjunctival tissue was deemed more relevant for the expression of inflammatory markers. Expression of all serine proteases was increased in untreated dry eye, with the greatest effect on tryptase (p = 0.036). In contrast to conjunctival tissue, corneal tissue of untreated dry eye displayed a significant increase in PAR2 expression (p = 0.033). uPAR expression was increased in corneal tissue, but this result was only statistically significant for UAMC-00050 treated animals. Tissue of UAMC-00050 and vehicle treated animals showed no decline in expression of these genes, compared to untreated animals (Table 1). It did however, suggest the involvement of tryptase, PAR2 and uPAR in DES in this animal model. It must be noted that UAMC-00050 is not expected to have an effect on the expression of serine proteases, as UAMC-00050 inhibits protein activity, rather than expression. Furthermore, a lack of activation could result in upregulation of the PAR2 and uPAR receptors.

### Gelatin zymography

**Figure 7:**
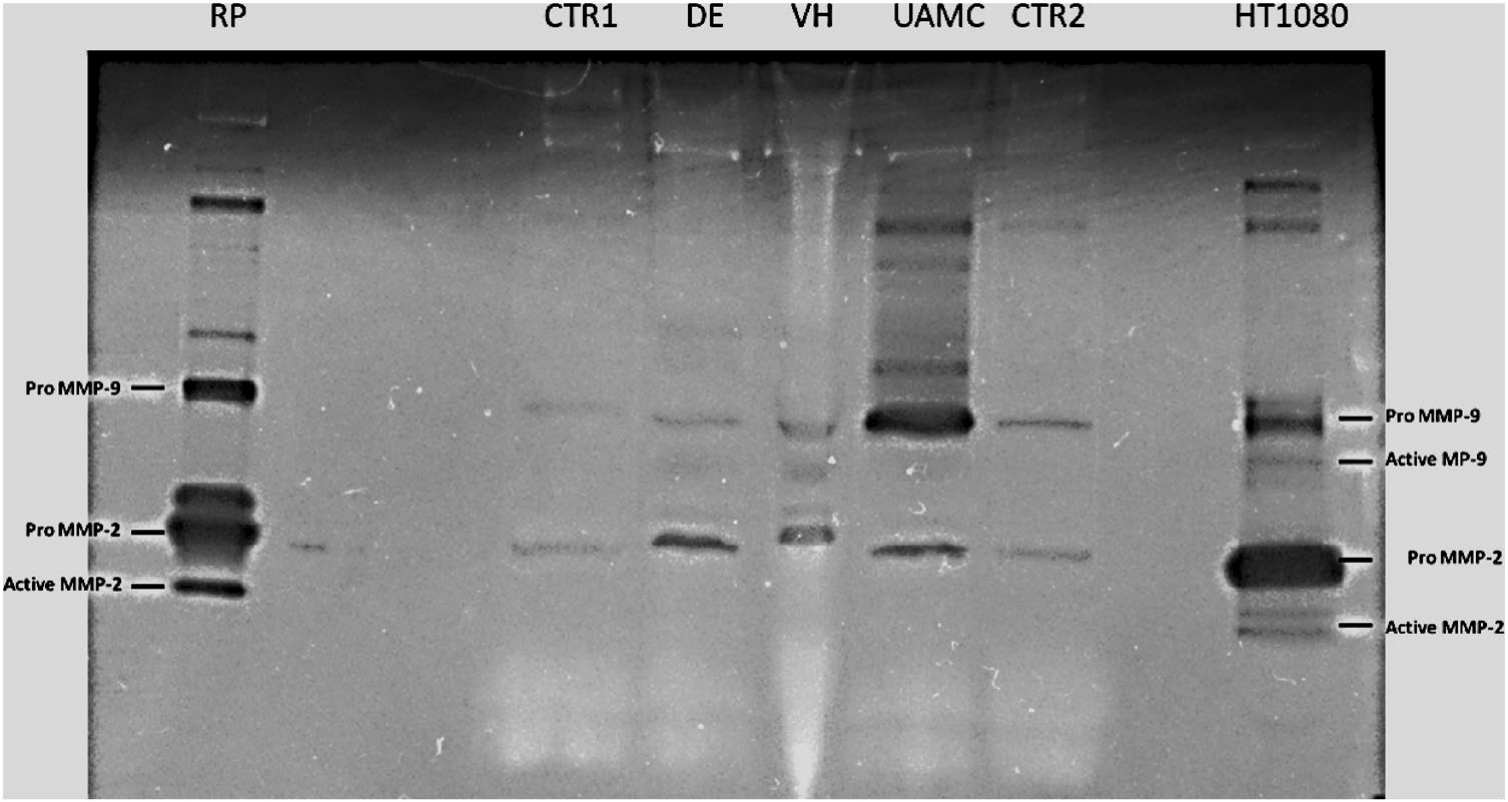
Representative gel zymography. RP: rat plasma, CTR1-2: healthy control tear fluid, DE: tear fluid from untreated rats with dry eye, VH: tear fluid from rats with dry eye treated with vehicle, UAMC: tear fluid from rats with dry eye treated with UAMC-00050, HT1080: medium from Human HT1080 cells. 5 µl sample loaded per lane. Samples from 1 pool of 6 animals for each group.

An accumulation of pro-MMP-9 could clearly be observed in tear fluid from rats with dry eye treated with UAMC-00050 (Fig. 7). Active MMP-9, an important biomarker of dry eye, was found in untreated dry eye and vehicle treated dry eye, but was not present in control and UAMC-00050 treated samples. Additionally, the presence of pro-MMP-2 was increased in tear samples from all animals with dry eye (treated and non-treated), compared to the control.

#### Stability of the formulation

**Figure 8:**
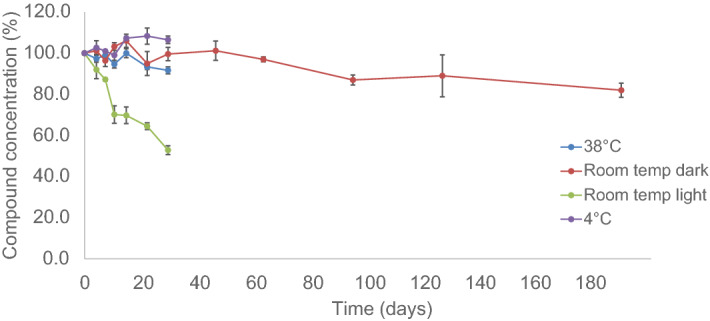
Quantitative analysis of the active agent in the formulation as a function of time (mean of 3 samples ± SD).

The quantitative analysis showed (Fig. 8) that light had a negative effect on the stability of UAMC-00050 in the formulation. Samples stored protected from light at elevated temperature (38 °C) as well as the samples stored at refrigerated temperature (4 °C) were found to be stable up to 30 days. At R.T., the assay value of the active ingredient in the formulation did not decrease below 85% after 180 days. Samples stored at R.T. exposed to light showed a rapid decrease in assay of the active compound in the formulation, as after 30 days only 52% of the initial amount was present. From these results, it was concluded that the formulation is stable when protected from light, even at an elevated storage temperature.

Initially, the samples had a pH of 4 ± 0.05 and an osmolality of 337 ± 1 mOsm kg^−1^. The osmolality of the formulations did not change significantly during the storage time of 28 days, regardless of the light conditions. The osmolality level of both samples remained constant during the stability screening at 335 ± 3 mOsm kg^−1^ (mean ± SD). Although the osmolality of the samples was slightly higher than the level of a healthy eye (around 290 mOsm kg^−1^), no irritation was observed during the ocular tolerance test. The pH measurement showed no change during the 28 days of storage (pH level 3.9 ± 0.1).

### Sterility

The sterility test showed that the formulation hindered the microbial growth below the concentration of 100 CFU of the micro-organisms, while at higher concentration this hindering effect was not observed. This effect was not present in the case of *Staphylococcus aureus*. As an antimicrobial effect was observed at low micro-organism concentration, the test product was filtered to remove the antimicrobial products and the filter was subsequently incubated in both digest medium (Fluid thioglycollate medium and Soya-bean casein) and the corresponding incubation temperature for 14 days. No microbial growth occurred, indicating complete sterility of the eye formulation.

## Discussion and conclusion

Treatment options for DES are currently very limited and as proteases have shown to be involved in various inflammatory settings, we investigated a potent inhibitor of serine proteases, the UAMC-00050 compound for treatment of DES.

In this study, no statistically significant effect of any treatment on tear volume was found. A reason for this could be the nature of the induction method in this animal model, i.e. the removal of the exorbital lacrimal gland. However, a positive trend of UAMC-00050 on tear volume was observed. The evaluation of this compound on another, non-aqueous-tear deficient dry eye model (e.g. an evaporative dry eye model), is justifiable to elucidate this effect further.

In four consecutive experiments, UAMC-00050 at 5 mM was the best overall treatment compared to the positive control CyA and the vehicle. UAMC-00050 (5 mM) treated animals were the only group with a statistically significant effect on tissue damage scores, starting from 9 days after the start of the experiment. Additionally, a significant difference in tissue damage scores was observed between UAMC-00050 (5 mM) and vehicle treated animals. UAMC-00050 (5 mM) was the only treatment with a statistically significant effect on both cytokines (IL-1α and TNF-α), measured in the tear fluid. No statistically significant difference in mean tear cytokine concentrations between UAMC-00050 (5 mM) and vehicle treated animals was observed however.

This distinct vehicle effect was only significant for TNF-α. This implies that tear substituents do succeed in lowering inflammatory markers in tear fluid. This effect did not extrapolate to reducing ocular surface damage, inflammatory cell infiltration in ocular tissue and MMP-9 activity in the tear fluid, in contrast to UAMC-00050. This suggests that the vehicle effect is predominantly due to dilution.

Treatment with UAMC-00050 at a lower dose (0.5 mM) also had a positive effect on the fluorescein score and IL-1α and TNF- α concentrations in tear fluid, but this effect declined after 3 weeks. This dose-dependent effect provides further evidence of a relationship between the therapeutic effect of the compound and specific targets.

Infiltration of both CD3^+^ and CD45^+^ cells was observed in tissue of animals with DES. Treatment with UAMC-00050 (5 mM) corresponded with a statistically relevant decline in inflammatory cells, in comparison to vehicle treated and untreated animals. This effect was most pronounced for CD3^+^ cells. Neutrophils are known to secrete a number of PAR2-activating serine proteases, responsible for extracellular matrix degradation and the promotion of an inflammatory milieu^44,45^. If the PAR2 response and subsequent downstream inflammatory impulse is inhibited, then the extracellular effects from serine proteases, secreted from neutrophils and other cell types, might be less severe. Furthermore, if through PAR2 inhibition, the initial inflammatory response of innate cells and proteases is reduced, less CD3^+^ T-cells are expected to be recruited to the inflamed tissue. Although not specifically investigated in this study, mast cells might contribute to the CD45^+^ cells. Mast cells are known to secrete tryptase and to activate PAR2 in inflammatory conditions^46^. These cells, initially only thought to contribute to allergic-inflammation, are now known for their effects in non-allergic inflammation. Moreover, mast cells secrete TNF-α and can act as APCs to recruit T-cells^46–48^. In future research we aim to include mast cell staining to better characterize the CD45^+^ cells involved in experimental dry eye, as this is currently a limitation to the research described in this paper.

Recent publications confirm the upregulation of PAR2 in ocular diseases, including DES^20,25,26^. PAR2 is highly expressed on inflammatory cells and its activation is related to the formation of a pro-inflammatory milieu and an inflammatory response^26^. PAR2 is expressed on endothelial and epithelial cells and can regulate cellular activity. PAR2 activation is able to induce MAPK signaling, including extracellular signal-regulated kinases (ERK)-1 & 2 and to a lesser extent P38 and JNK. NF-κβ-dependent gene expression of pro-inflammatory cytokines and the leukocyte adhesion molecule ICAM-1, are also initiated by PAR2 signaling^28^. Interestingly, activation of PAR2 by serine proteases plays an important role in innate and inflammatory responses of corneal infection and inflammation^25^. It has been reported that the serine protease inhibitor A3K has promising experimental in vivo results in a mouse and rabbit model for dry eye^29,49^. In addition, KLK13 has also recently been discovered as a potential target for DES^11^.

In untreated animals with DES, an increased IL-17A and IL-1α expression and CD3^+^ infiltration was reported in this study. Treatment with UAMC-00050 (5 mM) decreased these effects, suggesting an effect of the inhibitor on the recruitment and presence of Th17 cells. It has already been demonstrated that PAR2 signaling promotes DC trafficking to the lymph nodes and subsequent T-cell activation^50^. The observation of reduced IL-1α and TNF-α concentrations in tear fluid from UAMC-00050 treated rats might directly contribute to the presence of less immunocompetent cells in ocular tissue. This was demonstrated in the immunohistochemical analysis of conjunctival tissue and the preservation of ocular surface health through Na-fluorescein staining. The latter might be correlated with reduced serine protease and MMP-9 activity. Protecting the ocular surface from extracellular matrix degradation, cellular damage and subsequent cytokine production, can lead to a less severe inflammatory response as observed in this study.

Elevated tryptase gene expression was observed in animals with dry eyes in both conjunctival and corneal tissue. Treatment with UAMC-00050 did not lower the expression of this protease, even though it is expected to inhibit its activity. The same rationale could be used for PAR2 and uPAR because UAMC-00050 is also an inhibitor of the uPA active site. Both target genes displayed a slight elevation in expression in both tissues of animals with DES. Treatment with vehicle or UAMC-00050 did not alter their expression levels. This is in contrast to IL-1α and IL17A, where treatment did have an influence on the expression of these genes. This is further evidence that UAMC-00050 inhibits targets, responsible for the upregulation of both cytokines.

The accumulation of pro-MMP-9 in tear fluid from rats with dry eye and treated with UAMC-00050, provides evidence that through trypsin-like serine protease inhibition, activation of (pro)MMP-9 was also blocked. In untreated dry eye and vehicle treated dry eye, no Pro-MMP-9 accumulation was found. Moreover, in these samples, active MMP-9 was observed, while this was not the case for control tear fluid and tear fluid from rats, treated with UAMC-00050. Of note, MMP-9 activity was rather low in the dry eye and vehicle treated samples. Here, low sample volume and the fact that active MMPs are cleared very rapidly^51^ might be the reason for the modest expression.

The vehicle effect observed in this study can be attributed to dilution of the inflammatory markers of the residual tear fluid. Primarily because the vehicle effect in this study is most pronounced on cytokine concentration in the tears. However, in this study, the last administration of eye drops and tear collection are at least 14 h apart. The application of artificial tears twice a day, starting from the moment dry eye is induced, might be initially able to retard the onset of ocular inflammation. Especially when taking into account there were only 23 days between induction of dry eye and the end of the experiment. Moreover, in all experiments, relative humidity, which is directly proportional to tear film stability, was kept constant at 50%. It might be possible that these vehicle effects are less pronounced in long-term experiments and subsequent dry eye progression. Finally, it has to be considered that the vehicle (eye drops), in contrast to vehicles for other diseases, by itself has a therapeutic effect by dilution and lubrication.

In obtaining convincing results it was instrumental not only to find an effective active agent against DES, but also to develop a suitable eye-drop formulation with an excellent stability to be able to successfully administer the serine protease inhibitor.

The results of this study suggest the involvement of these serine proteases in DES and ocular inflammation. Moreover, a proof-of-concept was provided for trypsin-like serine protease inhibitors as a potential treatment for DES and ocular inflammation. Further experiments are needed to gain a better insight into the exact biochemical and immunological pathways involved in this process.

## Methods

### Animals

Female Wistar rats (200–300 g, Janvier, Roubaix, France) were kept under standard pathogen-free conditions^43^. Husbandry conditions: room temperature 20–25 °C, humidity 50–60% and a day–night cycle of 12 h light/12 h dark. Food and water were available ad libitum. All in vivo manipulations were approved by the Animal Ethical Committee of the University of Antwerp (2013-67) and are in accordance with the ARRIVE guidelines for the use of animals in ophthalmic and vision research.

### Anesthesia

To remove the exorbital lacrimal gland, animals were anesthetized with an intraperitoneal injection of 25 mg kg^−1^ ketamine (Anesketin, Eurovet, Bladel, Netherlands) and 2.5 mg kg^−1^ xylazine (Rompun, Bayer, Leverkusen, Germany)^43^. Routine manipulations (tear collection, fluorescein staining) were performed after induction with 5% isoflurane (Halocarbon, New Jersey, USA), followed by a maintenance dosage of 1.5%.

### Induction of dry eye

DES was induced by removal of the exorbital lacrimal gland, as described previously^43^. In brief, to remove the gland, a small incision was made beneath the right ear. The lacrimal glands of the left eye were kept intact. Progression of dry eye was monitored for 23 days.

### Measurement of aqueous tear production

A phenol red thread (Zone Quick, Menicon Co. Ltd, Nagoya, Japan) was placed in the lateral cantus of the conjunctival fornix for 15 s^43^. Absorption of tear fluid resulted in a color shift from yellow to red and tear distance was measured in millimeters.

### Evaluation of ocular surface damage by fluorescein staining

Sodium-fluorescein (1%, Sigma-Aldrich, Seelze, Germany) in phosphate buffered saline (PBS, Gibco by LifeTechnologies Europe, Gent, Belgium) was administered topically to the surface of the eye^43^. Eyes were rinsed after one minute with PBS and excess fluorescein was removed. The eye was photographed with a microscopic lens (Photo adapter 1.0 MC 80 DX—Axiovert 25 CA, Carl Zeiss AB, Göttingen, Germany) in a darkened room under cobalt blue light. Using the Oxford fluorescein grading scale, scores from 0 to 5 were given to each eye, depending on ocular staining intensity. Semi-quantitative scoring was done in a blind manner by three independent observers.

### Tear collection

Tear fluid was collected once a week with 10 µl capillaries (Blaubrand Intramark, Wertheim, Germany) connected with a flexible tube to a syringe)^43^. Immediately after collection, capillaries and tear fluid were stored at − 80 °C for immunological analyses.

### Tear fluid analyses

TNF-α and IL-1α concentrations in tear fluid were measured by flow cytometry (FACSarray, BD Biosciences), using a Cytometric Bead Array (CBA) according to the manufacturer’s protocol (Rat IL-1α and TNF-α CBA flex sets, BD Biosciences, Erembodegem Belgium)^43^.

### Tissue collection and preservation

At the end of the experiment, animals were euthanized by gradual CO_2_ overdose. Immediately after death, the cornea and palpebral and bulbar conjunctiva were removed and stored in the appropriate manner^43^. For qRT-PCR, tissue was placed in RNA-later (Sigma-Aldrich, Seelze, Germany) and stored at − 80 °C until RNA-extraction and subsequent RT-PCR-analysis. For immunohistochemistry, tissues were fixed in 4% paraformaldehyde for 24 h. The tissue was then transferred to a cryostat, frozen in optical cutting temperature (OCT) (Tissuetek, USA) and stored at − 80 °C.

### qRT-PCR

Total RNA from cornea and palpebral + bulbar conjunctiva was isolated using the RNeasy Plus Mini Kit (Qiagen, Venlo, The Netherlands)^43^. cDNA was generated during the qRT-PCR analysis, using the SensiFAST HI-ROX onestep PCR kit. Expression of PAR2, tryptase α/β, matriptase uPAR, IL-1α and IL-17A, relative to the endogenous reference genes ACTB (actin beta) and GAPDH (glyceraldehyde 3-phosphate dehydrogenase) was determined using TaqMan gene expression assays (Applied Biosystems, California, USA). (All primers from LifeTechnologies Europe, Gent, Belgium). RT-PCR reactions were set up in triplicate and were performed on a StepOnePlus Real-Time PCR system (Applied Biosystems, California, USA). Amplification conditions consisted of 12 min at 45 °C (reverse transcription), 2 min at 95 °C, 44 cycles of 20 s at 95 °C and 30 s at 60 °C.

### Immunohistochemistry

Immune cell infiltration in the palpebral conjunctiva was analyzed immunohistochemically^43^. The tissue was fixed in paraformaldehyde (for 24 h), frozen in optimal cutting temperature (OCT, TissueTek, Sakura Finetek, USA) and stored at − 80 °C. Histological cryo-sections of the inner eyelid (15 µm) were transferred to poly-l-lysine coated slides and incubated with 0.05% thimerosal, 0.01% NaN3, 0.1% bovine serum albumin, 1% triton-X100 and 10% normal horse Serum in PBS. The samples were incubated with anti-CD3 primary antibody or anti-CD45 primary antibody (Abcam, Cambridge, UK) followed by goat anti-rabbit CyTM3 (Jackson ImmunoResearch Europe Ltd, Suffolk, UK) or goat anti-rabbit fluorescein isothiocyanate (FITC) respectively. A nuclear counterstaining was applied using 4′,6-diamidino-2-phenylindole (DAPI, LifeTechnologies Europe). All slides were analyzed using a Zeiss observer Z1 AX10 fluorescence microscope (Carl Zeiss AB, Göttingen, Germany). Rabbit IgG polyclonal isotype control was included (Abcam, Cambridge, United Kingdom). Color balance (red) was slightly adjusted using ImageJ software to improve the visibility of the positive cells. Processing was applied equally across the entire image and to controls.

### Zymography

Sample preparation and gel zymography were executed as described previously^52^. In short, rat plasma and equilibrating buffer containing 0.5 M NaCl, 10 mM CaCl_2_ and 0.01% Tween-20 in TBS, were incubated with gelatin-conjugated sepharose beads (gelatin sepharose 4B, GE Healthcare Europe, Belgium) for 20 min at room temperature for affinity precipitation. The beads were rinsed and the gelatinases were eluted with 20 µl zymogram loading buffer (Novex tris glycine SDS sample buffer, Life technologies, Belgium) after which 15 µl was loaded onto the electrophoresis gel.

Fifteen µl of HT1080 sample, 10 µl of cell culture medium of HT1080 fibrosarcoma cells mixed with 5 µl sample buffer, was used as positive control. Five µl tear samples were diluted with 5 µl RNase-free water and 5 µl sample buffer. Ten µl molecular weight marker (ProSieve color protein marker, Lonza Switzerland) was included in the zymography.

Samples were loaded on a 10% gelatin gel (Life Technologies Europe, Belgium) and run for 90 min at 125 V with Tris–glycine SDS running buffer (Novex, Thermofisher, USA). The gels were subsequently incubated in zymogram renaturing buffer (Novex, Thermofisher, USA), rinsed in zymogram developing buffer (Novex, Thermofisher, USA) and incubated for 2 days at 37 °C. After staining with Coomassie blue, gels were destained for 2 h and imaged, using the ChemiDoc MP imaging system (Biorad, Belgium) and processed using Image lab (Biorad, Belgium) software.

### Compounds

**Figure 9:**
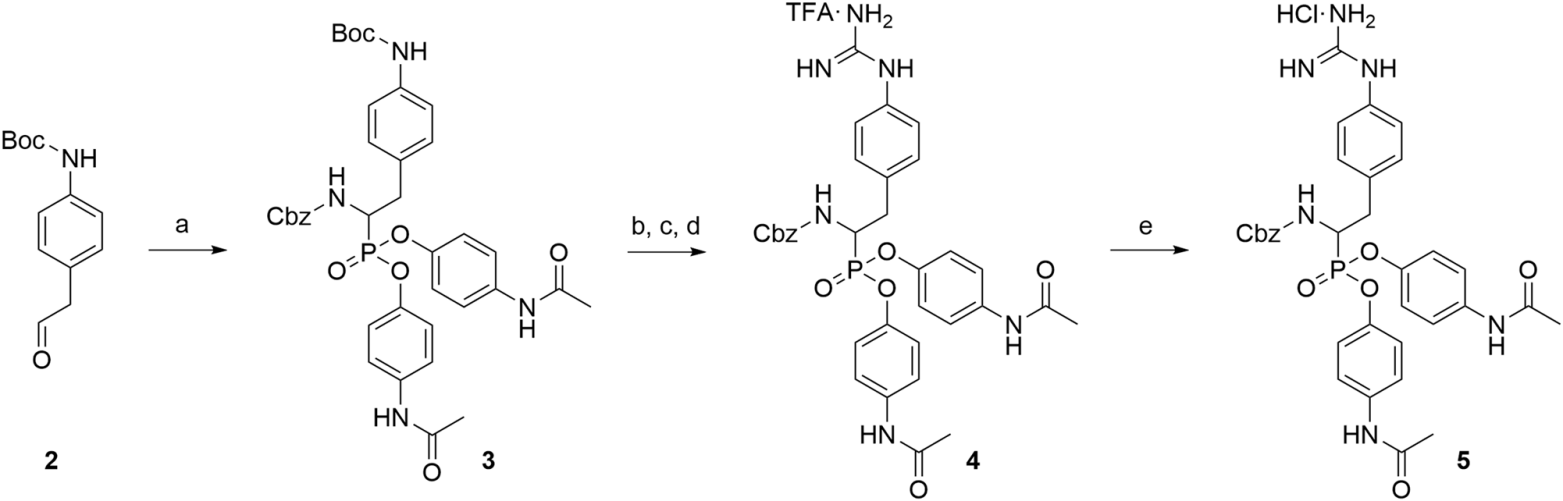
Reagents and conditions: (**a**) Tris(4-acetamidophenyl)-phosphite (fresh), CbzNH_2_, Cu(OTf)_2_, ACN, R.T., 16 h; (**b**) TFA (50% in DCM); (**c**) N,Nʹ-bis(tert-butoxycarbonyl)-1-guanylpyrazole, Et_3_N, DCM:ACN (2:1), R.T., 120 h; (**d**) TFA (50% in DCM); (**e**) Dowex 1X8 Cl^-^ form, EtOH:Water (2:1), R.T., 32 h.

The serine protease inhibitor UAMC-00050 was synthesized based on the pathway by Joossens et al*.*^31^. The synthesis route was adapted to a higher scale (≈ 1 g) by minor changes as reported in Fig. 9. The main variation was the addition of a last step, using an ion exchange resin, to obtain the final hydrochloride salt preferred for in vivo purposes, as briefly described below:

- **1-(4-(2-(((benzyloxy)carbonyl)amino)-2-(bis(4-acetamidophenoxy)phosphoryl)ethyl) phenyl)guanidinium trifluoroacetate (3)** was synthesized adapting the synthesis as described previously^31^.
- **1-(4-(2-(((benzyloxy)carbonyl)amino)-2-(bis(4-acetamidophenoxy)phosphoryl)ethyl) phenyl)guanidinium chloride (4)**.
- **1-(4-(2-(((benzyloxy)carbonyl)amino)-2-(bis(4-acetamidophenoxy)phosphoryl)ethyl) phenyl) guanidinium trifluoroacetate (3)** (1.2 g, 1.55 mmol) was dissolved in a mixture ethanol:water (2:1) and was stirred with Dowex 1X8 Cl- form (12.0 g) at room temperature (R.T.) for 32 h. The solution was then filtrated and the filtrate concentrated to yield 1-(4-(2-(((benzyloxy)carbonyl)amino)-2-(bis(4-acetamidophenoxy)phosphoryl)ethyl)-phenyl) guanidinium chloride (1.03 g, 1.48 mmol, 96% yield) as a white powder. ^1^H NMR (400 MHz, DMSO-*d*_*6*_) δ: 10.16 (s, *J* = 2.0 Hz, 2H), 10.04 (s, 1H), 8.23 (t, *J* = 14.0 Hz, 1H), 7.66–7.44 (m, 7H), 7.38 (d, *J* = 8.5 Hz, 2H), 7.34–7.23 (m, 3H), 7.23–7.04 (m, 8H), 4.97 (q, *J* = 12.5 Hz, 2H), 4.47 (ddd, *J* = 14.5, 12.5, 3.0 Hz, 1H), 3.32–3.17 (m, 1H), 3.07–2.93 (m, 1H), 2.04 (s, 6H). ^13^C NMR (101 MHz, DMSO- *d*_*6*_) δ: 168.3, 168.3, 156.1–155.8 (m), 145.1 (dd, *J* = 25.7, 10.0 Hz), 136.6 (d, *J* = 10.5 Hz), 135.2 (d, *J* = 17.5 Hz), 133.9, 130.4, 128.4, 127.9, 127.5, 127.1, 124.0, 120.9, 120.8, 120.6, 120.5, 120.2, 120.2, 65.7, 49.7 (d, *J* = 156.9 Hz), 33.6, 24.0. C/MS t_r_ = 1.29 min, 100%. MS (ESI) m/z 659.3 = [M + H]^+^.

### Formulation development

Different ophthalmologic formulations were evaluated. The initial formulation was based on a 5% (w/v) Povidon (PVP) ophthalmologic solution^53^, which contained 5% (w/v) PVP K30 (Mw 40 000, BASF, Ludwigshafen, Germany), 0.4% (w/v) sodium dihydrogen phosphate (Merck, Darmstadt, Germany), 1.6% (w/v) disodium hydrogen phosphate (Merck, Darmstadt, Germany), 0.9% (w/v) sodium chloride (Carl Roth, Karlsruhe, Germany), 0.02% (w/v) disodium ethylenediaminetetraacetic acid (Sigma-Aldrich, Steinheim, Germany), 0.1% (w/v) benzalkonium chloride (Fargon, Waregem, Belgium)) and 34.8 µg ml^−1^ UAMC-00050 which corresponds to a 50 µM concentration. All components of the formulation were dissolved consecutively in ultra-pure water without heating. This initial formulation did not meet the stability requirements, showing a significant decrease in UAMC-00050 assay from 50 to 10 µM in a period of 7 days. It became clear that the compound was more stable at acidic pH than at neutral or basic pH. Thus, an improved formulation, the boric acid-benzalkonium chloride solution^54^ was selected with a pH of 4 which is still acceptable for eye formulations. This standard solution consisted of 5% (w/v) PVP K30, 4% (w/v) boric acid, 0.2% (w/v) sodium ethylenediaminetetraacetic acid and 0.02% (w/v) benzalkonium chloride (BAC). The composition of the vehicle was optimized by evaluating the stability and ocular tolerance and the following vehicle formulation was chosen without the use of BAC: 20 mg ml^−1^ boric acid, 25 mg ml^−1^ PVP K30 and 1 mg ml^−1^ Na_2_EDTA with a pH of 4.

The selected formulation was prepared by first dissolving all components of the vehicle in demineralised water, after which the appropriate amount of UAMC-00050 was added. The solution was filtered through a 0.22 µm pore size syringe filter into sterile Eppendorf tubes, which were closed firmly and covered with parafilm (Santa Cruz Biotechnology, inc, Dallas, United States).

All activities were performed under aseptic conditions, in a horizontal laminar air flow cabinet (S@femate 1.8, BioAir, Milan, Italy).

### Stability of the formulation

To evaluate the stability, 4 batches were prepared in triplicate and stored at following conditions; 4 °C protected from light for 30 days, elevated temperature (38 °C) protected from light for 30 days, R.T. without light protection for up to 30 days and R.T. protected from light for up to 180 days. Samples were taken for analysis on day 0, 3, 7, 10, 14, 30, (60, 90, 120 and 180). Quantitative determination of the compound in the solutions was performed by reversed phase high pressure liquid chromatography (HPLC). The HPLC-system consisted of a pump (Shimadzu LC-20AT), a DAD-detector (Shimadzu SPD-M20A), a degasser (Shimadzu DGU-20A5), an auto-sampler (Shimadzu SIL-20A) and a column (GraceSmart RP18 Column 150 × 4.6 mm 5u 120A). The total flow rate was set at 1.0 ml min^−1^ and the mobile phase consisted of acetonitrile (ACN)/acetic acid (1% (v/v)) with a gradient starting from 5% (v/v) ACN up to 95% (v/v) ACN. The injection volume was 20 μl and detection was performed at 260 nm. The integration of the peak area was performed using LC Postrun Analysis (Shimadzu) and the concentration was calculated with reference to an external calibration curve.

The pH of the samples was determined with a calibrated pH meter (HI 5221, Hanna Instruments, Limena, Italy).

Physical stability was assessed by visual inspection and osmolality measurements which were performed by a Micro Osmometer Model 3300 (Advanced Instruments, Norwood, MA, USA) using the freeze-point depression method. Osmolality and pH values were determined on day 0, 7, 14, 21 and 28.

### Sterility of the formulation

The sterility was tested on day 0 according to the guidelines of the European Pharmacopeia^55^. Fluid thioglycollate medium and Soya-bean casein were used as digest medium. Both aerobic (*Staphylococcus aureus* and *Pseudomonas aeruginosa*) and anaerobic (*Clostridium sporogenesis*) bacteria and fungi (*Candida albicans*) were tested as micro-organisms. Care was taken to respect the different growth conditions of all micro-organisms. The samples were incubated for 14 days at 35 °C. If no microbial growth could be observed visually, the samples were considered sterile.

### Statistical analyses

#### Tear volume, ocular surface damage, tear cytokine concentrations

The change of the respective parameter over time, and the influence of the treatments, was modelled using linear mixed models. Concentration was entered as dependent variable. Baseline concentration, treatment and time (categorical) were entered as independent variables, plus the interaction between time and treatment. This latter term models whether the difference in concentration between the treatments, is equal across time points.

If there was no interaction between time points and treatment, a statistical evaluation of the mean effect of a treatment on the duration of the whole experiment was possible. If there was an interaction between time points and treatments, meaning the effect of the treatment is different for each time point, no universal statement was possible. If the latter was the case, statistics were only applied per time point.

Subsequently, the dataset was split to study the main effects of time and treatment separately. The data were split by time point. Within each time point, we analyzed if there was a significant difference in concentration between the treatments using a one-way ANOVA. In case the effect of treatment was significant, we carried out a *post-hoc* analysis with Tukey correction for multiple testing.

The data were split by treatment. Within each treatment, a test for a significant difference in concentration between time points using a linear mixed model was conducted. In case the effect of time was significant, a *post-hoc* analysis with Tukey correction for multiple testing was carried out (Supplementary Information).

#### qRT-PCR and immunohistochemistry

Statistical analyses were performed with GraphPad using non-parametric tests. Different experimental groups were compared by a One-way ANOVA test. Only when p-values ≤ 0.05 were obtained, *post-hoc* pairwise comparisons by means of Mann Whitney U was performed. *p < 0.05, **p < 0.01, ***p < 0.001.

### Experimental design

The potent inhibitor (UAMC-00050) was selected for in vivo testing in 4 different experiments, totaling 18 animals/group. In these experiments, (Evaluation of UAMC-00050), tear volume and corneal tissue damage were evaluated once a week. Tear collection occurred twice a week. Animals were treated with CyA (2x/day), UAMC-00050/vehicle (2×/day) for 24 consecutive days. Mean values of 4 experiments (with 4–6 animals per group for each experiment) ± SD are displayed in the results section below. All preparations were administered in a blind manner (color coded).

An additional 24-day experiment, employing untreated dry eye, control, vehicle and UAMC-00050 treated animals (with N = 6 for each group), was conducted to obtain tear samples for zymography and the preliminary study regarding PAR-activation. In this experiment, tear sampling occurred three times a week and fluorescein staining and tear volume measurements were excluded.

#### *Evaluation of UAMC-00050*

Control: The left eye of every animal in all experimental groups functioned as control.
Dry eye: Removal of the right exorbital lacrimal gland. Animals received no treatment.
CyA: Removal of the right exorbital lacrimal gland. Animals received 2.5 µL CyA 0.05% twice a day.
UAMC-00050 5 mM: Removal of the right exorbital lacrimal gland. Animals received 2.5 µL UAMC-00050 at 5 mM twice a day.
UAMC-00050 0.5 mM: Removal of the right exorbital lacrimal gland. Animals received 2.5 µL UAMC-00050 at 0.5 mM twice a day.
Vehicle: Removal of the right exorbital lacrimal gland. Animals received 2.5 µL vehicle twice a day.

## Supplementary information

Supplementary Information.

## Data Availability

All data described in this manuscript and all details of methods described will be made readily available upon requests made to the corresponding author.
