# Supplementary material for: A novel serine protease inhibitor as potential treatment for dry eye syndrome and ocular inflammation
Source: Sci Rep. 2020 Oct 14;10:17268. doi: 10.1038/s41598-020-74159-w (PMC7560718; doi:10.1038/s41598-020-74159-w)

**Supplementary information**

# "A novel serine protease inhibitor as potential treatment for dry eye syndrome and ocular inflammation"

UAMC-00050 as potential treatment for dry eye syndrome

Cedric Joossen[1*], Adrienn Baán[21], Carlos Moreno-Cinos[3], Jurgen Joossens[3], Nathalie Cools[4], Ellen Lanckacker[1], Lieve Moons[5], Kim Lemmens[5], Anne-Marie Lambeir[6], Erik Fransen[7], Peter Delputte[1], Guy Caljon[1], Pieter Van Der Veken[3], Louis Maes[1], Ingrid De Meester[6], Filip Kiekens[2], Koen Augustyns[3], **Paul Cos[1]

[1]Faculty of Pharmaceutical, Biomedical and Veterinary Sciences: Laboratory of Microbiology, Parasitology and Hygiene (LMPH), University of Antwerp, Universiteitsplein 1, 2610 Wilrijk, Belgium

[2]Faculty of Pharmaceutical, Biomedical and Veterinary sciences: Laboratory of Pharmaceutical Technology and Biopharmacy, University of Antwerp, Universiteitsplein 1, 2610 Wilrijk, Belgium

[3]Faculty of Pharmaceutical, Biomedical and Veterinary Sciences: Laboratory of Medicinal Chemistry, University of Antwerp, Universiteitsplein 1, 2610 Wilrijk, Belgium

[4] Faculty of Medicine and Health Sciences: Laboratory of Experimental Hematology, Vaccine & Infectious Disease Institute (VAXINFECTIO), University of Antwerp, Universiteitsplein 1, 2610 Wilrijk, Belgium

[5]Faculty of Biology: Neural Circuit Development and Regeneration Research Group, University of Leuven, Naamsestraat 61, Belgium

[6]Faculty of Pharmaceutical, Biomedical and Veterinary Sciences: Laboratory of Medical Biochemistry, University of Antwerp, Universiteitsplein 1, 2610 Wilrijk, Belgium

[7]Faculty of Pharmaceutical, Biomedical and Veterinary Sciences: Laboratory of Medical Genetics, University of Antwerp, Universiteitsplein 1, 2610 Wilrijk, Belgium

1. **Tear volume in relation to time**

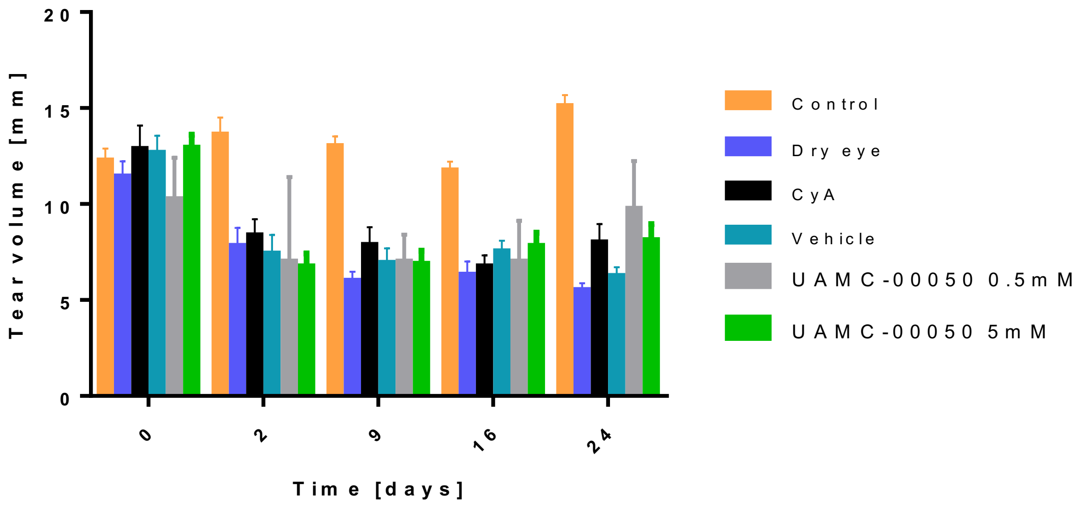


Mean tear volume [mm] in relation to time of control eyes, untreated dry eyes, CyA, vehicle and UAMC-00050 0.5 and 5 mM treated eyes. Data represent mean values ± SD of 4 experiments (Ntot=18) except for UAMC-00050 0.5 mM, where N=5 (1 experiment).

| Time-point [days] | Control | | | Dry eye | | | CyA | | | Vehicle | | | UAMC00050 0.5mM | | | UAMC00050 5mM | | |
|---|---|---|---|---|---|---|---|---|---|---|---|---|---|---|---|---|---|---|
| | Mean [mm] | SD | N | Mean [mm] | SD | N | Mean [mm] | SD | N | Mean [mm] | SD | N | Mean [mm] | SD | N | Mean [mm] | SD | N |
| 0 | 12.3 | 2.6 | 18 | 11.4 | 3.3 | 18.0 | 12.9 | 5.2 | 18 | 12.7 | 3.8 | 18 | 10.3 | 2.2 | 5 | 12.9 | 2.8 | 18 |
| 2 | 13.6 | 3.7 | 18 | 7.8 | 3.9 | 18.0 | 8.4 | 3.6 | 18 | 7.4 | 4.2 | 18 | 7.0 | 4.4 | 5 | 6.8 | 2.8 | 18 |
| 9 | 13.0 | 2.1 | 18 | 6.0 | 2.0 | 18.0 | 7.9 | 3.9 | 18 | 6.9 | 3.2 | 18 | 7.0 | 1.4 | 5 | 6.9 | 2.9 | 18 |
| 16 | 11.8 | 1.9 | 18 | 6.3 | 2.8 | 18.0 | 6.8 | 2.4 | 18 | 7.5 | 2.3 | 18 | 7.0 | 2.1 | 5 | 7.8 | 2.9 | 18 |
| 24 | 15.1 | 2.4 | 18 | 5.5 | 1.5 | 18.0 | 8.0 | 4.1 | 18 | 6.3 | 1.9 | 18 | 9.8 | 2.5 | 5 | 8.1 | 3.5 | 18 |

Mean tear volume [mm] data table of control eyes, untreated dry eyes, CyA, vehicle and UAMC-00050 0.5 and 5 mM treated eyes.

**2. IL-1α concentration in relation to time**

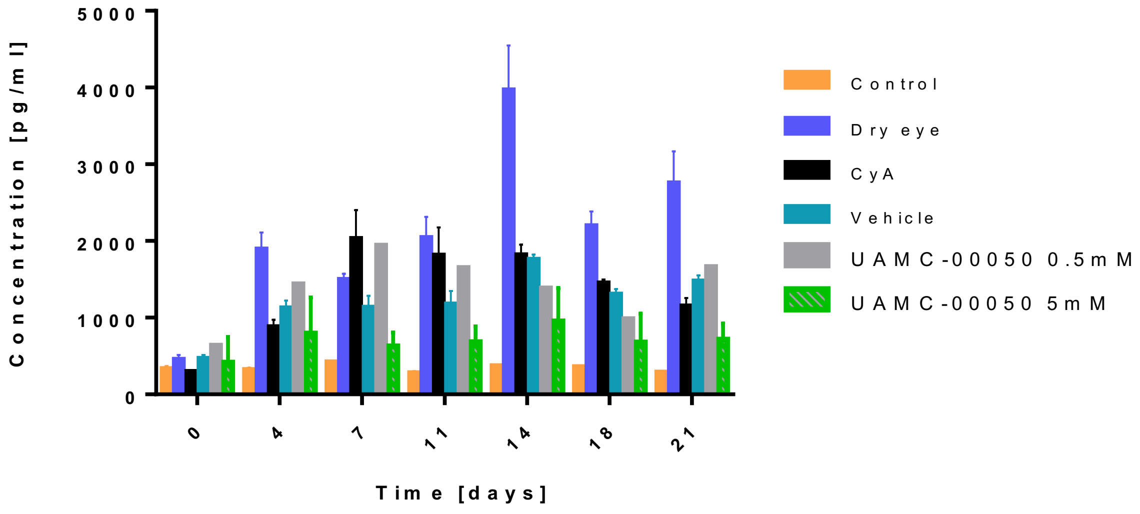


Mean IL-1α concentration [pg/ml] in relation to time of control eyes, untreated dry eyes, Restasis, vehicle and UAMC-00050 0.5 and 5 mM treated eyes. Data represent mean values ± SD of 4 experiments (Ntot=18) except for UAMC-00050 0.5 mM, where N=5 (1 experiment).

**3. TNF-α concentration in relation to time**

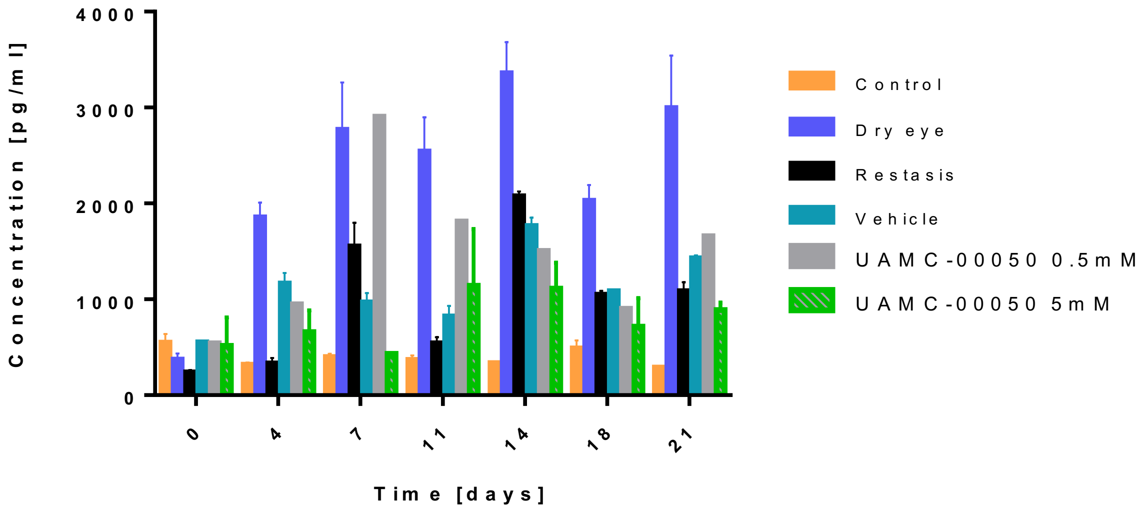


TNF-α concentration [pg/ml] in relation to time of control eyes, untreated dry eyes, Restasis, vehicle and UAMC-00050 0.5 and 5 mM treated eyes. Data represent mean values ± SD of 4 experiments (Ntot=18) except for UAMC-00050 0.5 mM, where N=5 (1 experiment).

.

**4. Mean CD45+ cell counts (results Immunohistochemistry)**

| Control | Dry eye | UAMC-00050 | Vehicle |
|---|---|---|---|
| 2.42 ± 3.29 | 48 ± 9,9*** | 22 ± 12** | 45 ± 2.5** |

Table 1: Mean CD45+ cell count ±SD per 500 DAPI stained cells in palpebral conjunctival tissue from untreated rats with dry eye, control animals and UAMC-00050 and vehicle treated animals *: $p<0.05$ **: $p<0.01$ ***: $p<0.001$ versus control.

**5. Mean CD3+ cell counts (results Immunohistochemistry)**

| Control | Dry eye | UAMC-00050 | Vehicle |
|---|---|---|---|
| 1.8 ± 2,7 | 30 ± 3.0** | 7.4 ± 4.1 | 28 ± 3.3* |

Table 2: Mean CD3+ cell count ±SD per 500 DAPI stained cells in palpebral conjunctival tissue from untreated rats with dry eye, control animals and UAMC-00050 and vehicle treated animals *: $p<0.05$ **: $p<0.01$ versus control.

**6. Stability of pH and osmolality during 28 days of stability assessment at two different conditions**

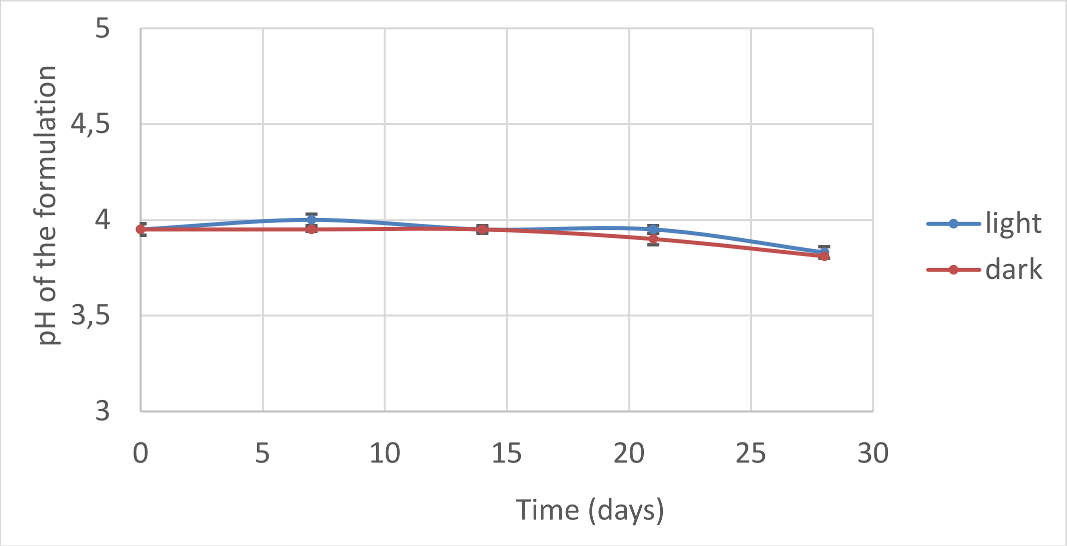


pH in function of time of samples stored at room temperature in presence and in absence of light up to 28 days (mean of 3 ± SD)

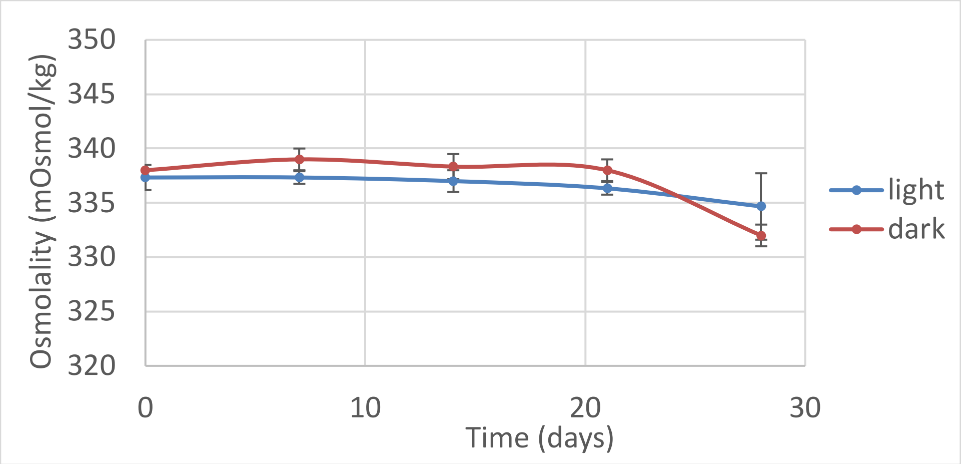


Osmolality in function of time of samples stored at room temperature in presence and in absence of light up to 28 days (mean of 3 ± SD)

### 7. Synthetic Procedure of the UAMC-00050 in a higher scale

Unless otherwise stated, laboratory reagent grade solvents were used. Reagents were obtained from Sigma-Aldrich, Acros Organics, or Fluorochem and were used without further purification. Characterization of all compounds was done with $^{1}H$ and $^{13}C$ NMR and mass spectrometry. $^{1}H$ and $^{13}C$ NMR spectra were recorded on a 400 MHz Bruker Avance III Nanobay spectrometer with Ultrashield and analyzed by use of MestReNova analytical chemistry software. Chemical shifts are in ppm, and coupling constants are in hertz (Hz). ES mass spectra were obtained from an Esquire 3000 plus ion trap mass spectrometer from Bruker Daltonics. Purities were determined with two diverse HPLC systems based either on mass determination or on UV detection. A Waters acquity UPLC system coupled to a Waters SQD ESI mass spectrometer was used both in combination with a Waters TUV detector. Water (A) and ACN (B) were used as eluents. Waters Acquity UPLC BEH C18 1.7 µm, 2.1 mm × 50 mm column was used. Solvent A consisted of water with 0.1% formic acid. Solvent B consisted of acetonitrile with 0.1% formic acid. Method I involved the following: 0.15 min 95% A, 5% B, then in 1.85 min from 95% A, 5% B to 95% B, 5% A, then 0.25 min (0.350 mL/min), 95% B, 5% A. The wavelength for UV detection was 254 nm. Where necessary, flash purification was performed on a Biotage ISOLERA One flash system equipped with an internal variable dual wavelength diode array detector (200–400 nm). For normal phase purifications SNAP cartridges (10–340 g, flow rate of 10–100 mL/min) were used. Dry sample loading was done by self-packing samplet cartridges using silica and Celite 545, respectively, for normal and reversed phase purifications.

The following sections comprise the synthetic procedures and analytical data for all compounds reported in this manuscript.

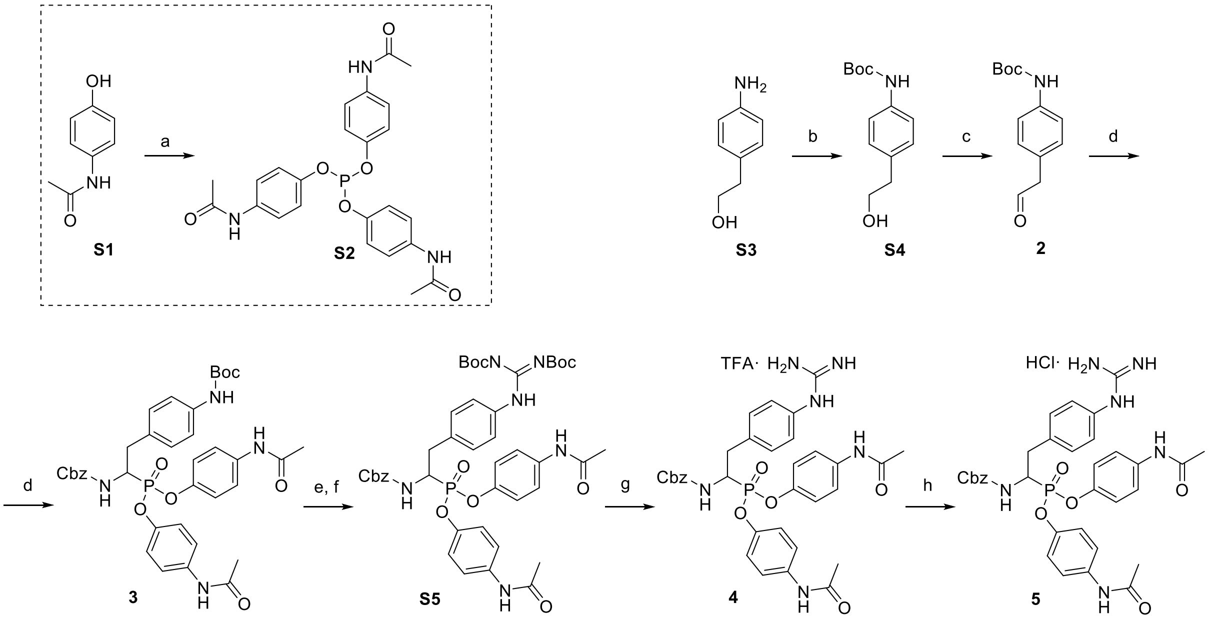

**Scheme 2:** *Reagents and conditions:* (a) $PCl_3$, $Et_3N$, THF, 0 ºC, 90 min; (b) $Boc_2O$, $Et_3N$, dioxane, r.t.; (c) Dess-Martin periodinane, DCM, 0-25 ºC, r.t.; (d) Int. S2, $CbzNH_2$, $Cu(OTf)_2$, ACN, r.t., 16 h; (e) TFA (50% in DCM); (f) *N*,*N*'-bis(*tert*-butoxycarbonyl)-1-guanylpyrazole, $Et_3N$, DCM:ACN (2:1), r.t., 120 h; (g) TFA (50% in DCM); (h) Dowex 1X8 $Cl^-$ form, EtOH:Water (2:1).

**Tris(4-acetamidophenyl) phosphite (S2)**

Dry $Et_3N$ (3,5 eq) was added dropwise to a stirred solution of phosphorustrichloride (1 eq) and acetaminophen (3,5 eq) in THF (Volume for 0,2 M solution of $PCl_3$) at 0 °C. When the addition was complete the mixture was stirred at 0 °C for 15 min, and then the reaction mixture was allowed to slowly reach r.t. and was stirred for 90 min. The reaction mixture was filtered through a small pad of celite, and the filtrate was concentrated under reduced pressure (the temperature of the water bath was kept not above 40 °C in order to avoid degradation. The crude product was derived as a white foam tris(4-acetamidophenyl) phosphite (98% yield), which was used immediatedly for the next synthetic step. Analytical characterization for compound **S2** was consistent with the previously reported data· (Joossen et al., 2007)

***Tert*-butyl (4-(2-hydroxyethyl)phenyl)carbamate (S4)**

To a solution of *p*-aminobenzylalcohol (1 eq) in dioxane (Volume for 0,4 M solution of *p*-aminobenzylalcohol) were added $Et_3N$ (3 eq) and di-*tert*-butyldicarbonate (1,1 eq) was added portionwise. The mixture was stirred at r.t. for 16 h. The solution was concentrated in vacuo. EtOAc was added and washed with 2N HCl, saturated $NaHCO_3$ and brine solution. The organic layer was dried over $Na_2SO_4$ and concentrated to yield *tert*-butyl (4-(2-hydroxyethyl)phenyl)carbamate (97% yield) as a brown oil that was used for the next synthetic step with no further purification. Analytical characterization for compound **S4** was consistent with the previously reported data (Joossen et al., 2007).

***Tert*-butyl (4-(2-oxoethyl)phenyl)carbamate (2)**

*Tert*-butyl 4-(hydroxymethyl)phenylcarbamate (**S4**) (1 eq) was added to a solution of Dess-Martin periodinane (1,1 eq) in DCM (Volume for 0,3 M solution of **S4**) at 0 °C. The solution was allowed to warm to r.t. and was stirred for 1 h at this temperature. The resulting solution was poured into a vigorously stirred saturated $NaHCO_3$ and $Na_2S_2O_3$ solution for 1 h. The organic layer was separated and washed with brine and dried over $Na_2SO_4$. The solvent was evaporated and the crude was purified by flash column chromatography ($SiO_2$, EtOAc in Heptane, 0/100 to 100/0). The desired fractions were collected and concentrated to yield *tert*-butyl (4-(2-oxoethyl)phenyl)carbamate (65% yield). Analytical characterization for compound **1** was consistent with the previously reported data· (Joossen et al., 2007)

**1-(4-(2-(((benzyloxy)carbonyl)amino)-2-(bis(4-acetamidophenoxy)phosphoryl)ethyl)phenyl)guanidinium 2,2,2-trifluoroacetate (3)**

*Tert*-butyl 2-(4-(2-oxoethyl)phenyl)carbamate (2) (1 eq), O-benzylcarbamate (1 eq) and tris(4-acetamidophenyl) phosphite (1 eq) were dissolved in ACN (Volume for 0.15 M solution of **1**). Then, copper(II) trifluoromethanesulfonate (0,1 eq) was added and the mixture was stirred at r.t. for 16 h. Then the mixture was evaporated and the crude was purified by flash column chromatography ($SiO_2$, EtOAc in Heptane, 50/50 to 100/0 followed by MeOH in DCM, 3/97 to 6/94). The desired fractions were collected and concentrated to yield 1-(4-(2-(((benzyloxy)carbonyl)amino)-2-(bis(4-acetamidophenoxy)phosphoryl)ethyl)-phenyl)guanidinium 2,2,2-trifluoroacetate (39% yield) as an off-white solid. Analytical characterization for compound **2** was consistent with the previously reported data (Joossen et al., 2007).

**Diphenyl 1-(Benzyloxycarbonylamino)-2-(4-(N,N'-Bis(*tert*-butyloxycarbonyl)guanidinophenyl)-ethanephosphonate (S5)**

Compound **3** was dissolved in 50% trifluoroacetic acid in DCM. After stirring for 3 h at r.t. the solvent was evaporated. The crude oil was washed with cold $Et_2O$ to obtain the deprotected aniline quantitatively, which was used for the next step with no further purification.

A mixture of this deprotected aniline (1 eq), *N*,*N'*-bis(*tert*-butyloxycarbonyl-1-guanyl-pyrazole (1 eq), and $Et_3N$ (3 eq) in DCM and ACN (1 mL of each/100 mg of **S5**) was stirred at r.t. for 5 days. The solvent was evaporated and the residue was dissolved in EtOAc and washed with 1 N HCl, saturated solution of $NaHCO_3$ and brine. The organic layer was dried over $Na_2SO_4$. The solvent was evaporated and the crude product was purified by flash column chromatography ($SiO_2$, EtOAc in Heptane, 0/100 to 80/20). The desired fractions were collected and concentrated to yield diphenyl 1-(benzyloxycarbonylamino)-2-(4-(*N*,*N'*-bis(*tert*-butyloxycarbonyl) guanidinophenyl)-ethanephosphonate (50% yield) as an off-white solid. Analytical characterization for compound **S5** was consistent with the previously reported data (Joossen et al., 2007).

**1-(4-(2-(((benzyloxy)carbonyl)amino)-2-(bis(4-acetamidophenoxy)phosphoryl)ethyl)phenyl)guanidinium trifluoroacetate (4)**

Compound **S5** was dissolved in 50% trifluoroacetic acid in DCM. After stirring for 3 h at r.t. the solvent was evaporated. The crude oil was washed with cold $Et_2O$ to obtain 1-(4-(2-(((benzyloxy)carbonyl)amino)-2-(bis(4-acetamidophenoxy)phosphoryl)ethyl)phenyl)guanidinium trifluoroacetate (98% yield) as a white solid**.** Analytical characterization for compound **3** was consistent with the previously reported data (Joossen et al., 2007).

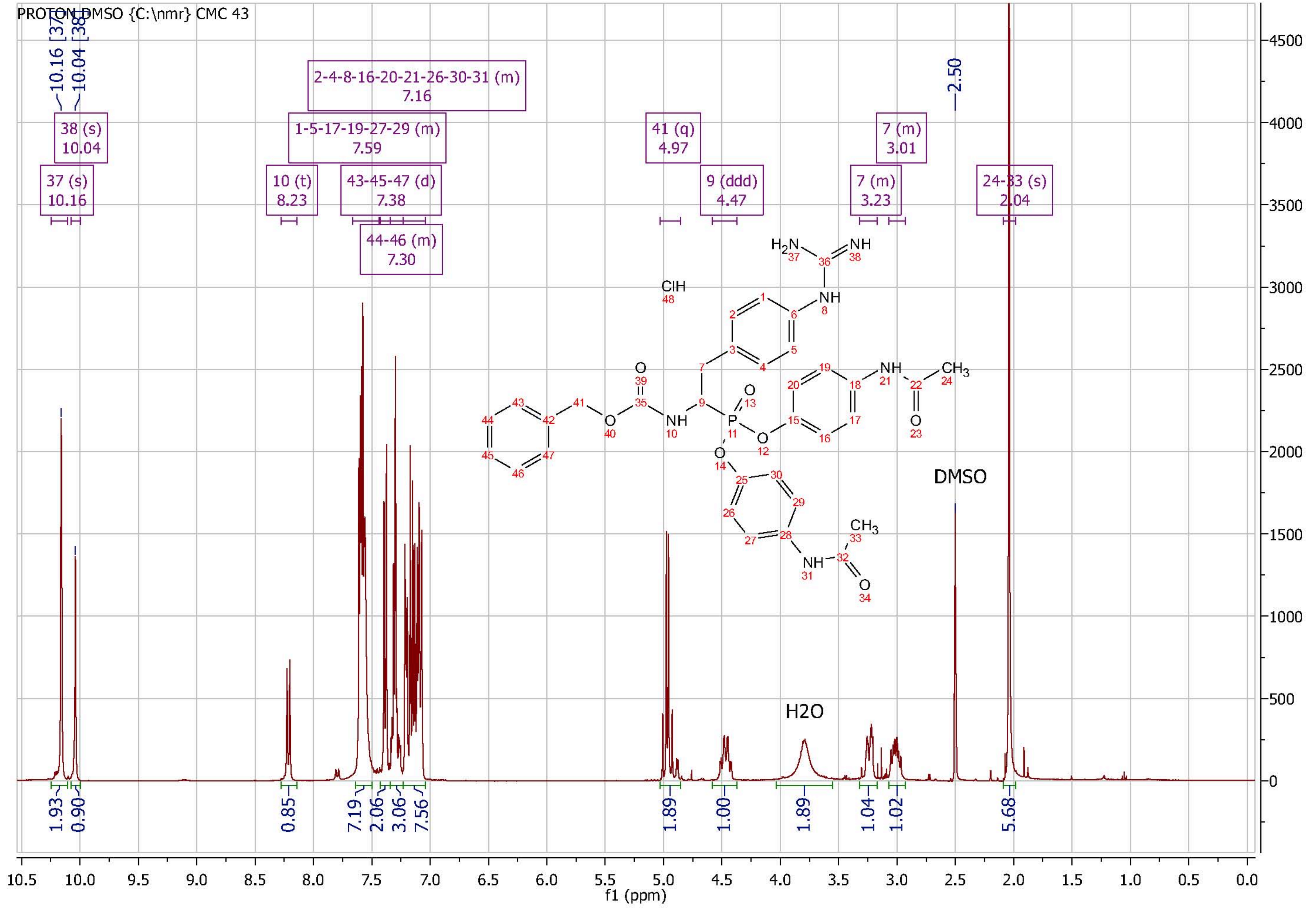
PROTON DMSO {C:\nmr} CMC 43
10.16 [37]
10.04 [38]
2-4-8-16-20-21-26-30-31 (m) 7.16
1-5-17-19-27-29 (m) 7.59
38 (s) 10.04
37 (s) 10.16
10 (t) 8.23
43-45-47 (d) 7.38
44-46 (m) 7.30
41 (q) 4.97
9 (ddd) 4.47
7 (m) 3.01
7 (m) 3.23
24-33 (s) 2.04
2.50
DMSO
H2O
1.93
0.90
0.85
7.19
2.06
3.06
7.56
1.89
1.00
1.89
1.04
1.02
5.68
f1 (ppm)

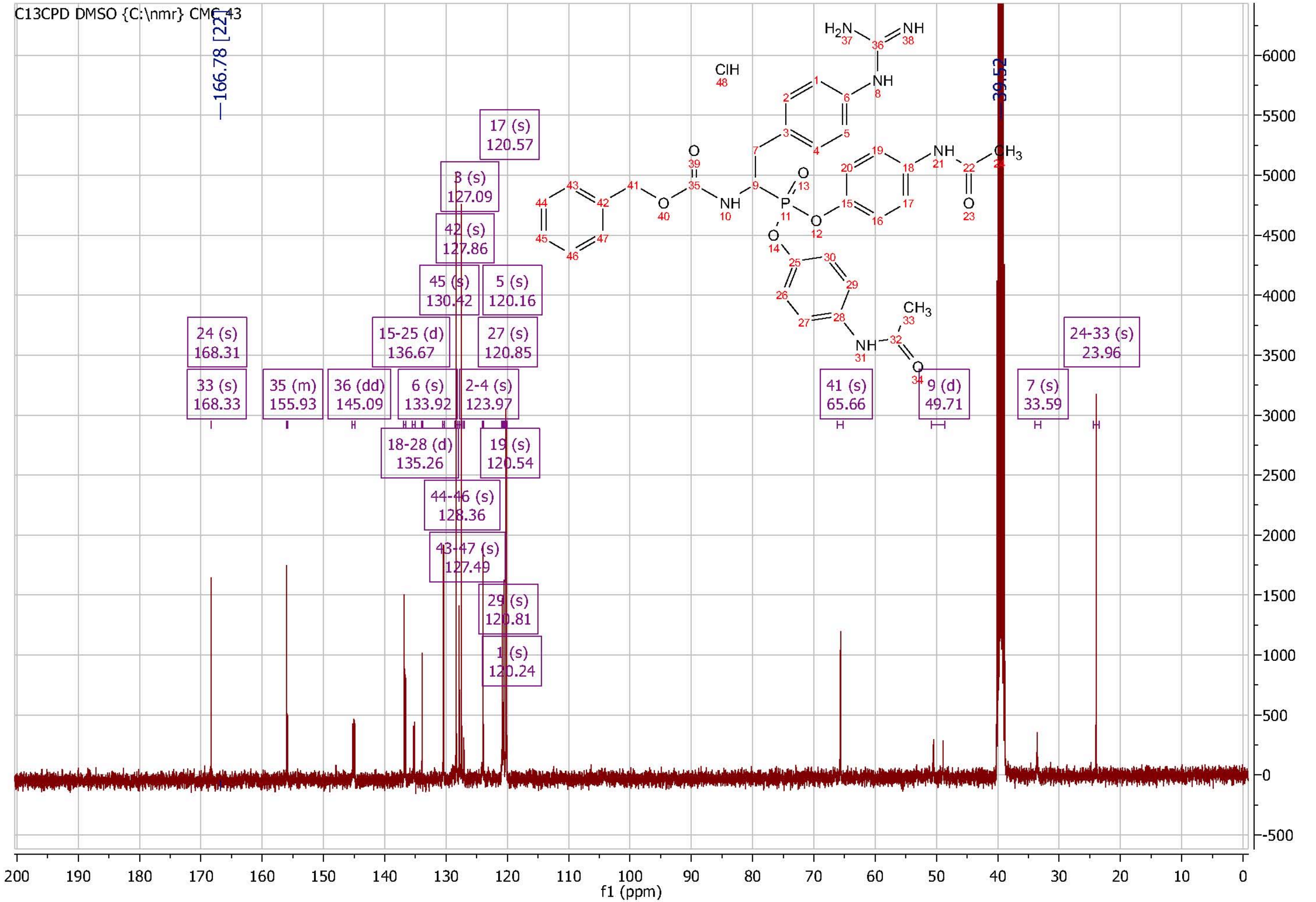
C13CPD DMSO {C:\nmr} CMC 43
166.78 [22]
39.52
17 (s) 120.57
3 (s) 127.09
42 (s) 127.86
45 (s) 130.42
5 (s) 120.16
24 (s) 168.31
15-25 (d) 136.67
27 (s) 120.85
24-33 (s) 23.96
33 (s) 168.33
35 (m) 155.93
36 (dd) 145.09
6 (s) 133.92
2-4 (s) 123.97
41 (s) 65.66
9 (d) 49.71
7 (s) 33.59
18-28 (d) 135.26
19 (s) 120.54
44-46 (s) 128.36
43-47 (s) 127.49
29 (s) 120.81
1 (s) 120.24
ClH
f1 (ppm)

**Openlynx Report - Carlos Moreno**

Sample: 1 | Vial:1:25 | ID:UAMC-00050-dil-CMC
File:UAMC-00050-dil-CMC | Date:20-Apr-2017 | Time:09:59:17
Description:

Printed: Thu Apr 20 10:05:06 2017

**Sample Report:**

**Sample 1 Vial 1:25 ID UAMC-00050-dil-CMC File UAMC-00050-dil-CMC Date 20-Apr-2017 Time 09:59:17 Description**

1: MS ES+ :BPI Smooth (SG, 2x2) 1.1e+007

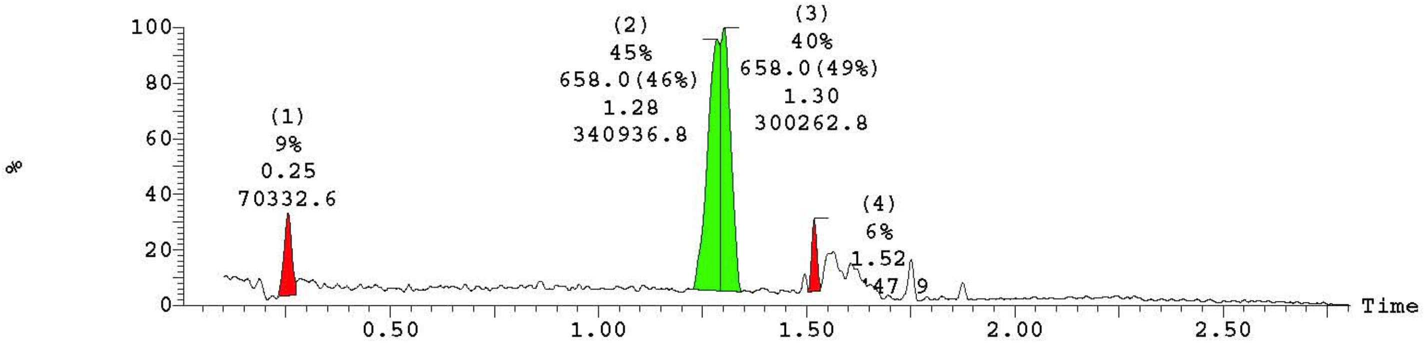


| Peak Number | Compound | Time | AreaAbs | Area % Total | Width | Height | Mass Found |
|---|---|---|---|---|---|---|---|
| 1 | | 0.25 | 7e+004 | 9.34 | 0 | 3e+006 | |
| 2 | Found | 1.28 | 3e+005 | 45.28 | 0 | 1e+007 | 658.00 |
| 3 | Found | 1.30 | 3e+005 | 39.88 | 0 | 1e+007 | 658.00 |
| 4 | | 1.52 | 4e+004 | 5.50 | 0 | 3e+006 | |

2: MS ES- :BPI Smooth (Mn, 2x3) 7.0e+005

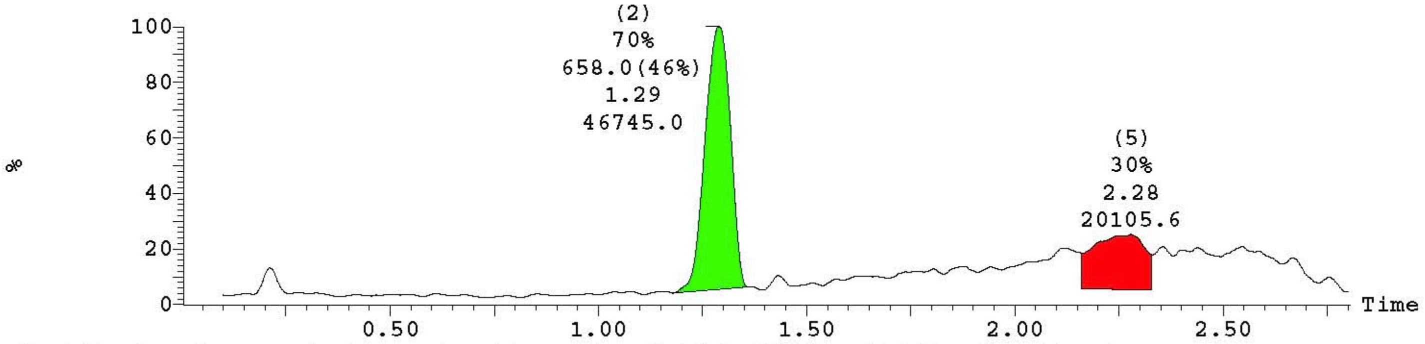


| Peak Number | Compound | Time | AreaAbs | Area % Total | Width | Height | Mass Found |
|---|---|---|---|---|---|---|---|
| 2 | Found | 1.29 | 5e+004 | 69.92 | 0 | 7e+005 | 658.00 |
| 5 | | 2.28 | 2e+004 | 30.08 | 0 | 1e+005 | |

3: UV Detector: TIC 3.234e-1
Range: 3.235e-1

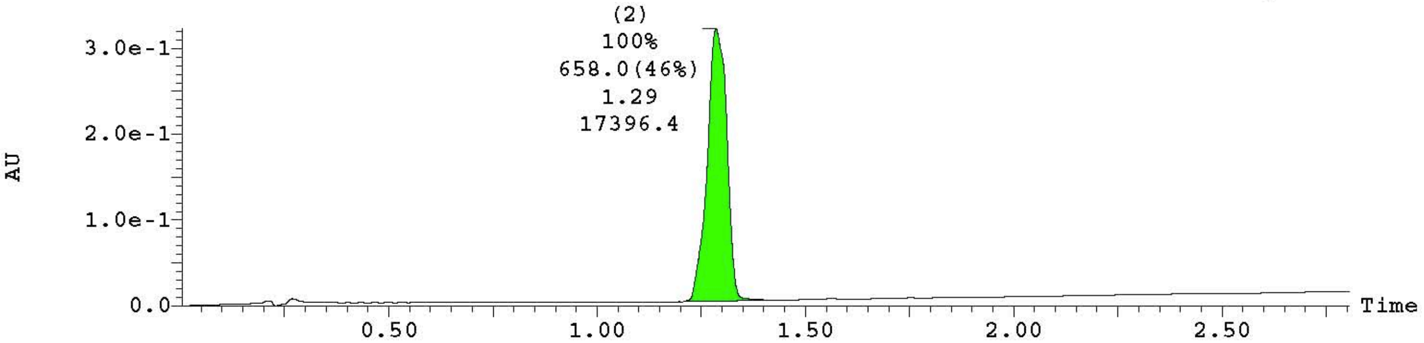


| Peak Number | Compound | Time | AreaAbs | Area % Total | Width | Height | Mass Found |
|---|---|---|---|---|---|---|---|
| 2 | Found | 1.29 | 2e+004 | 100.00 | 0 | 3e+005 | 658.00 |

Sample: 1 | Vial:1:25 | ID:UAMC-00050-dil-CMC
File:UAMC-00050-dil-CMC | Date:20-Apr-2017 | Time:09:59:17
Description:

Printed: Thu Apr 20 10:05:06 2017

**Sample Report (continued):**

| Peak ID | Compound | Time | Mass Found |
|---|---|---|---|
| 1 | | 0.25 | |

1:(Time: 0.25) Combine (64:70-(56:58+75:77)) 1:MS ES+ 2.4e+006

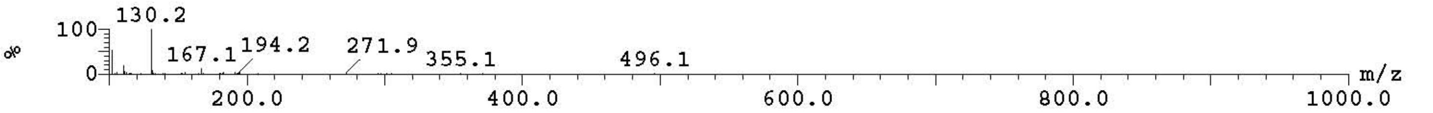


| Peak ID | Compound | Time | Mass Found |
|---|---|---|---|
| 2 | Found | 1.28 | 659.00,681.00 |

2:(Time: 1.29) Combine (334:339-(307:309+372:375)) 1:MS ES+ 1.0e+007

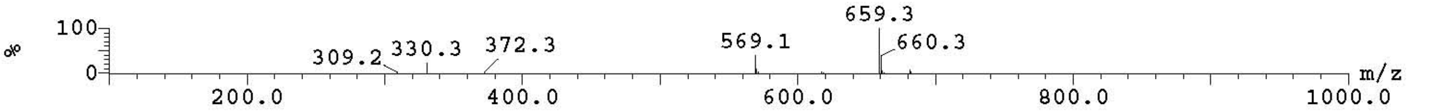


| Peak ID | Compound | Time | Mass Found |
|---|---|---|---|
| 3 | Found | 1.30 | 659.00,681.00 |

3:(Time: 1.30) Combine (338:344-(333:335+354:356)) 1:MS ES+ 5.1e+006

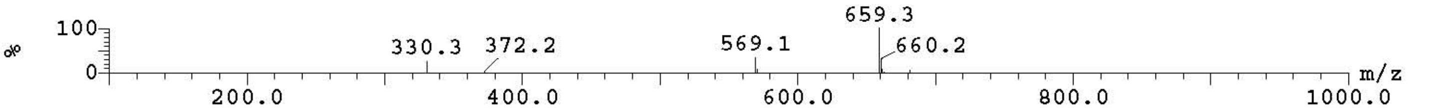


| Peak ID | Compound | Time | Mass Found |
|---|---|---|---|
| 4 | | 1.52 | |

4:(Time: 1.52) Combine (394:400-(388:390+404:406)) 1:MS ES+ 1.7e+006

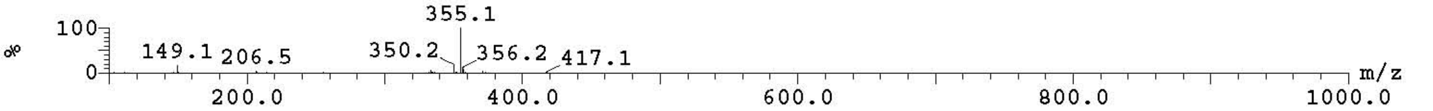


| Peak ID | Compound | Time | Mass Found |
|---|---|---|---|
| 2 | | 1.28 | |

2:(Time: 1.29) Combine (333:338-(306:309+371:374)) 2:MS ES- 7.3e+005

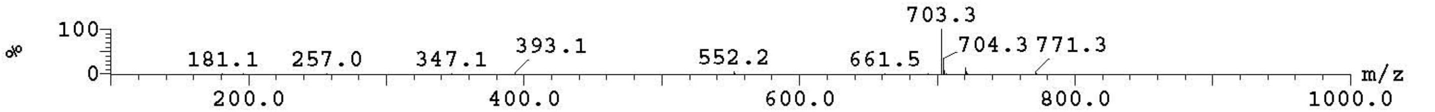


| Peak ID | Compound | Time | Mass Found |
|---|---|---|---|
| 5 | | 2.28 | |

5:(Time: 2.28) Combine (592:598-(559:561+611:613)) 2:MS ES- 2.2e+004

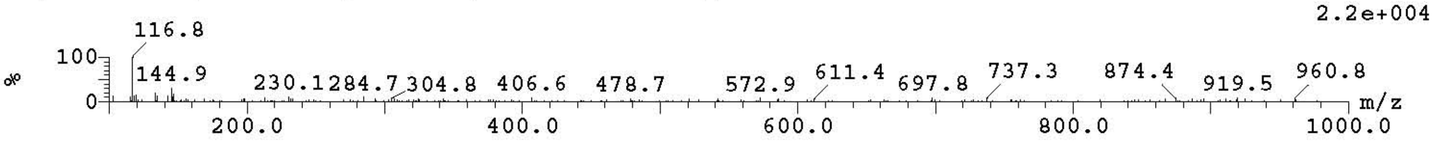

Supplement: Supplementary file 1 — Supplementary Information. [file 41598_2020_74159_MOESM1_ESM.pdf]
